# Epigenetic therapy of novel tumour suppressor ZAR1 and its cancer biomarker function

**DOI:** 10.1186/s13148-019-0774-2

**Published:** 2019-12-04

**Authors:** Verena Deutschmeyer, Janina Breuer, Sara K. Walesch, Anna M. Sokol, Johannes Graumann, Marek Bartkuhn, Thomas Boettger, Oliver Rossbach, Antje M. Richter

**Affiliations:** 1 Institute for Genetics, University of Giessen, 35392 Giessen, Germany; 2 Institute for Biochemistry, University of Giessen, 35392 Giessen, Germany; 3 Scientific Service Group Biomolecular Mass Spectrometry, Max-Planck Institute for Heart and Lung Research, 61231 Bad Nauheim, Germany; 4 The German Centre for Cardiovascular Research (DZHK), Partner Site Rhine-Main, Max-Planck Institute for Heart and Lung Research, 61231 Bad Nauheim, Germany; 5 Institute for Bioinformatics, University of Giessen, 35392 Giessen, Germany; 6 Max-Planck Institute for Heart and Lung Research, 61231 Bad Nauheim, Germany

**Keywords:** Cancer biomarker, ZAR1, Tumour suppressor, DNA methylation, Epigenetics, p53, Zinc finger, Epigenetic editing, CRISPR-Cas9

## Abstract

**Background:**

Cancer still is one of the leading causes of death and its death toll is predicted to rise further. We identified earlier the potential tumour suppressor zygote arrest 1 (ZAR1) to play a role in lung carcinogenesis through its epigenetic inactivation.

**Results:**

We are the first to report that *ZAR1* is epigenetically inactivated not only in lung cancer but also across cancer types, and *ZAR1* methylation occurs across its complete CpG island. *ZAR1* hypermethylation significantly correlates with its expression reduction in cancers. We are also the first to report that *ZAR1* methylation and expression reduction are of clinical importance as a prognostic marker for lung cancer and kidney cancer. We further established that the carboxy (C)-terminally present zinc-finger of ZAR1 is relevant for its tumour suppression function and its protein partner binding associated with the mRNA/ribosomal network. Global gene expression profiling supported ZAR1's role in cell cycle arrest and p53 signalling pathway, and we could show that ZAR1 growth suppression was in part p53 dependent. Using the CRISPR-dCas9 tools, we were able to prove that epigenetic editing and reactivation of *ZAR1* is possible in cancer cell lines.

**Conclusion:**

ZAR1 is a novel cancer biomarker for lung and kidney, which is epigenetically silenced in various cancers by DNA hypermethylation. ZAR1 exerts its tumour suppressive function in part through p53 and through its zinc-finger domain. Epigenetic therapy can reactivate the *ZAR1* tumour suppressor in cancer.

## Background

Cancer remains a devastating disease with 17 million new cases and 9.6 million deaths each year worldwide, as well as an expected continued rise of cases [1]. The total economic cost of cancer was estimated to be US$ 1.16 trillion [2], and only 1.4% of this staggering number is spent on cancer research [3]. Lung cancer remains the leading cause of cancer death by far [4], and we published that ZAR1 is a novel tumour suppressor in lung cancer [5]. Zygote arrest 1 (ZAR1) was initially reported to be a maternal-effect gene critical for oocyte to embryo transition in mouse [6]; however, we and others reported that its expression is not only limited to the oocyte but also found further tissues. *ZAR1* was reported to be expressed in porcine and bovine brain and testis [7], bovine heart and muscle [8], human lung [5], and rabbit lung [9]. Human ZAR1 locates on chromosome 4 (4p11) and harbours a large 1.5 kb CpG island (CGI; Additional file 1: Figure S1a). CpG islands are genomic regions defined by the enrichment of CpG dinucleotides [10]. ZAR1 codes for a 1275 nt transcript (4 exons) and a 424 aa protein with a carboxy (C)-terminal zinc-finger (CpG plot, NCBI, and UCSC genome browser; Additional file 1: Figure S1a). In the context of cancer, evidence has been growing for a role of ZAR1, even though early reports were in part contradicting. In melanoma, the methylation of exon 1 was reported, but *ZAR1* was said to be overexpressed in some hypermethylated melanoma cell lines [11]. In brain tumours and neuroblastoma, non-promoter methylation was reported [12, 13], as was its absence in hypermethylated glioma cell lines [12]. In hypermethylated neuroblastoma, however, expression of *ZAR1* was detected and indicated that ZAR1 knockdown promotes differentiation in neuroblastoma cells [13]. Intragenic *ZAR1* methylation decreased in high-grade vs. low-grade tumours of the bladder [14]. In hepatitis C virus, positive liver cancer *ZAR1* was reported to be methylated in exon 1 [15]. In cervical cancer, ZAR1 was methylated vs. normal epithelia [16]. With the present work, we report ZAR1 as a cancer biomarker and also elucidate its role in human cancer using state-of-the-art methylation sequencing, transcriptomic approaches, mass spectrometry for the identification of interacting partners, and epigenetic reactivation by CRISPR-dCas9.

## Results

### ZAR1 is a lung and kidney cancer biomarker

Our focus is the exciting and novel role of ZAR1 as a tumour suppressor in humans. *ZAR1* is differentially expressed across human tissues (testis, colon, kidney, lung, skin, and brain; Additional file 1: Figure S1b) and not restricted to the ovary (set 1 for comparability). Exploring a possible ZAR1 function in cancer, we found that *ZAR1* expression (*n* = 917, CCLE Cancer Cell Line Encyclopedia) significantly correlated with genes that carry the GO-terms ‘regulation of RNA metabolic processes’, ‘cell communication’, ‘signal transduction’, ‘cell–cell signalling’, ‘anatomical structure development’, and ‘embryonic morphogenesis’ (Additional file 1: Figure S1c). We earlier reported that *ZAR1* is epigenetically regulated in lung cancer [5]. Here, we add evidence for *ZAR1* hypermethylation in further cancer types (Additional file 1: Figure S2a, b). ZAR1 is methylated in various cell lines, in all germ cell lines (*n* = 14), in half of the malignant melanoma (*n* = 4) and kidney cancer cell lines (*n* = 4), in all mamma (*n* = 3), and 90% of brain cancer cell lines (glioblastoma; *n* = 8). In ovarian carcinoma, *ZAR1* hypermethylation increases from 15% in primary tumours (*n* = 20) to 67% in cancer cell lines (*n* = 6), whereas the controls were unmethylated (Additional file 1: Figure S3a, b). Quantification of *ZAR1* methylation revealed a methylation threshold at 20% (Additional file 1: Figure S3c).

**Fig. 1:**
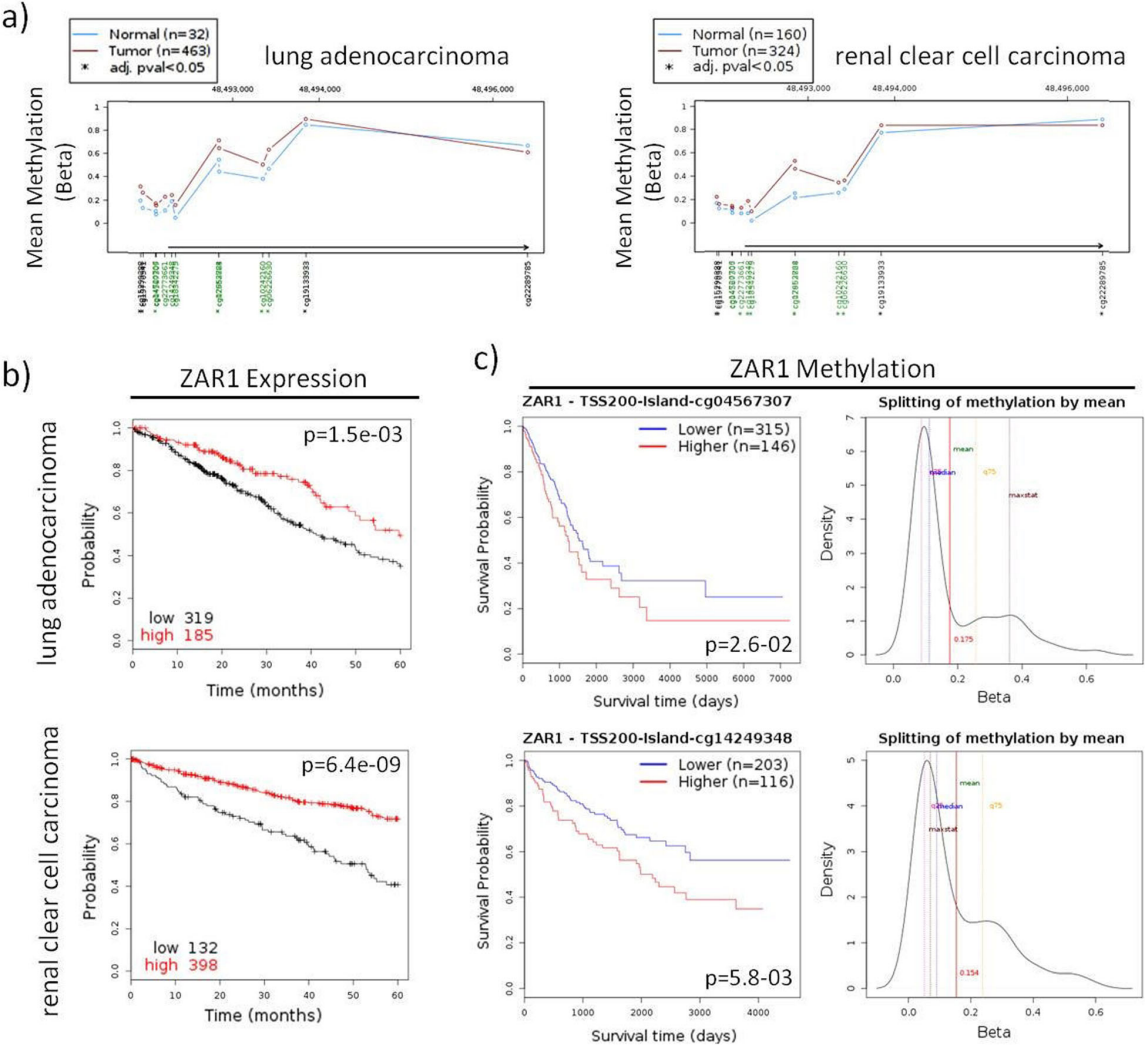
Inactivation of *ZAR1* is associated with decreased survival of lung and kidney cancer patients. **a** *ZAR1* is methylated in lung adenocarcinoma and renal clear cell carcinoma patients across the CGI (TCGA; 450k methylation array infinium chip; beta value; green, CGI; *, significance; red, tumour; blue, normal samples). **b** *ZAR1* expression-dependent survival in lung adenocarcinoma and renal clear cell carcinoma patients (5 years; red, high *ZAR1* expression; black, low *ZAR1* expression). **c** *ZAR1* methylation-dependent survival of lung adenocarcinoma and renal clear cell carcinoma patients (red, high *ZAR1* methylation; blue, low *ZAR1* methylation). Right graph shows methylation levels (beta value) split by mean as red vertical line

We next studied the complete ZAR1 CGI (Additional file 1: Figure S4a). Across cancer cell lines, the expression of *ZAR1* is significantly reduced in comparison to normal tissues (Additional file 1: Figure S4b, f; *p* = 2.4e-203). *ZAR1* methylation increases from tumour tissues to cancer cell lines in comparison to normal tissues (representative CpGs, Additional file 1: Figure S4c, d; *p* = 1.1e-35). This methylation change is observed across the complete CGI covered by 450k array probes (including N-shore Additional file 1: Figure S4e). We found that ZAR1 is expressed in the human kidney and lung (Additional file 1: Figure S1b) [5] and significantly hypermethylated in its CGI in lung adenocarcinoma and renal clear cell carcinoma vs. normal controls (Fig. 1a). Cancer patient survival was decreased with low *ZAR1* levels by the Kaplan–Meier Plot for lung and kidney cancer (Fig. 1b). Five-year survival probability of lung cancer patients was 35% with *low ZAR1* but 49% for *high ZAR1* group. Kidney cancer survival in *low ZAR1* group was 41% vs. 71% in *high ZAR1* group. Accordingly, *ZAR1* hypermethylation correlated with reduced patient survival in lung and kidney cancer (Fig. 1c). Methylation threshold was 17.5% for lung cancer and 15.4% for kidney cancer. The patients for ZAR1 expression in Fig. 1b and ZAR1 methylation in Fig. 1c are not matching samples (TCGA and 450K array methylation data, see methods for further information). The represented CpGs are cg045673007 in lung cancer and cg1424948 in renal cancer, which were most highly differentially methylated for the patient subgroups. This suggests that several CpGs might be analysed for ZAR1 methylation in function of the tumour of interest. In summary, *ZAR1* methylation and expression reduction are putative cancer biomarkers and targets for cancer prognostics.

### ZAR1 is highly conserved and an mRNA binding protein

**Fig. 2:**
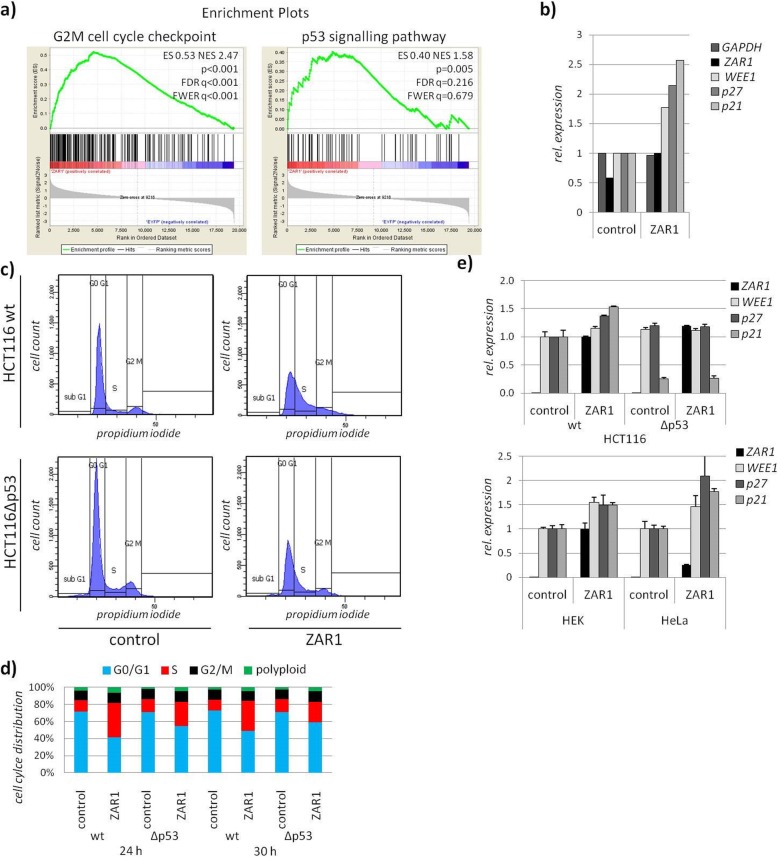
ZAR1 blocks cell cycle progression, partially through p53. **a** Enrichment plots from RNA microarray of ZAR1 overexpression reveals significant association with G2M checkpoint and the p53 pathway by GSEA analysis in HCT116. **b** According expression of *GAPDH*, *ZAR1*, *WEE1*, *p27*, and *p21* from array upon ZAR1 overexpression. **c** Overexpression of ZAR1 (EYFP tagged) in HCT116wt and HCT116Δp53 cells and subsequent cell cycle analysis by flow cytometry and propidium iodide staining. Gating to subG1, G0G1, S, and G2M phases is shown. **d** According quantification for 24 and 30 h overexpression of ZAR1 in HCT116 cells is calculated. **e** Altered RNA expression analysis upon ZAR1 or control reexpression is measured by quantitative RT-PCR and normalised to GAPDH for *ZAR1*, *WEE1*, *p21*, and *p27* in HCT116wt and HCT116Δp53 (upper) as well as in HEK and HeLa (lower)

*ZAR1* is in part conserved amongst vertebrates (Additional file 1: Figure S5a) and human, mouse, and xenopus. ZAR1 share a certain homology with high identity of human/mouse 91%, human/xenopus 96%, and mouse/xenopus 90% zinc-finger (analysed using *blastp*, *NCBI*, Additional file 1: Figure S5b). The high homology is especially observed across the C terminus (320-424aa human ZAR1). ZAR1 predicted phosphorylation/ubiquitination sites are conserved between mouse and human, which are also found C-terminally (Additional file 1: Figures S5b, S3a), where a highly conserved zinc-finger is present (by definition of human ZAR1 327aa-408aa). A secondary structure prediction of ZAR1 showed ordered α-helixes and β-strands in overlap with the C-terminal zinc-finger and the regions of higher homology (Additional file 1: Figure S5c, using [17]). Modelling ZAR1 (Additional file 1: Figure S5d; using *SWISS-MODEL* [18]) satisfactorily predicted the zinc-finger and zinc ion ligand. The genomic organisation of ZAR1 is also similar in mouse and human. Human *ZAR1* CGI is 1.5 kb (Additional file 1: Figures S1a, S4a), contains 68% G + C and an observed vs. expected CpG ratio of 0.98 (*UCSC*). The mouse *Zar1* CGI is 850 bp and also present across the promoter and first exon. It contains 71% G and C and the ratio of observed vs. expected CpG is >0.8 (*UCSC*). These genomic *ZAR1* characteristics are in accordance with the CpG island definition: length >200 bp, average of G and C >50%, and an observed vs. expected CpG >0.6 [19]. ZAR1 contains an atypical zinc-binding, (3CxxC, PF13695, *Pfam* [20]) with three pairs of CxxC motifs representing a multiple zinc-binding region. One pair of cysteines is associated with a highly conserved histidine residue (Additional file 1: Figure S5c). The only functional data on ZAR1 derived from xenopus, so far. In xenopus Zar1 was observed to bind to Wee1 mRNA and the translation regulation was zinc-finger dependent [21]. It was further reported that the Wee1 3’UTR contained a translation control sequence (TCS) consensus (A/U)UU(A/G)UCU regulating its translation [22]. We tested if human ZAR1 is also an mRNA binding protein assuming a certain degree of functional overlap from xenopus with human/mammalian ZAR1. We predicted ZAR1 potential TCS/ binding sites in human mRNA 3’UTRs (Additional file 1: Figure S6a). Potential ZAR1 binding motifs occur at the following rates in mRNA 3’UTRs: 2 in *n* = 1226, 3 in *n* = 121, and 4 in *n* = 8. *WEE1* contains the motifs UUUGUCU at position 559, UUUAUCU at 988 and a duplicate of AUUGUCU at 1043 and 1156 of its 1164 bp 3’UTR. In *WEE2* the motif UUUAUCU is found at 525 and 592 of its 951 bp 3’UTR. One duplicate motif is found in each *WEE*. We investigated the occurrence of duplicate motifs within a 200-bp sliding window across mRNA 3’UTRs. Numbers of mRNAs with at least duplicate motifs are: UUUAUCU in *n* = 171(WEE2), UUUGUCU in *n* = 175, AUUAUCU in *n* = 75, and AUUGUCU in *n* = 114 (WEE1). We aimed to understand through which motif human ZAR1 could bind to, e.g., WEE1 mRNA. We performed CLIP by precipitating ZAR1 and its bound RNA (Additional file 1: Figure S6b). Sequential RT-PCR covering the 3’UTR revealed the binding of ZAR1 to *WEE1* around the predicted duplicated TCS AUUGUCU (Additional file 1: Figure S6c). The ability of ZAR1 to interact with mature mRNA is consistent with its lack of nuclear localisation signals (using *cNLS mapper* [23]) and its exclusive cytosolic localisation (Additional file 1: Figure S7). Furthermore, a genome wide transcriptomic approach revealed that ZAR1 reexpression correlates with enriched ‘mRNA 3’end processing’ genes (Additional file 1: Figure S8). We also confirmed that ZAR1 regulates *WEE1* expression levels (microarray and RT-PCR) upon ZAR1 overexpression (Fig. 2b, e). Additionally, epigenetic reactivation of ZAR1 increased *WEE1* expression (Fig. 5d, f). Our findings are consistent with the role of the WEE1 kinase inhibiting CDK1, blocking mitosis and therefore negatively regulating cell cycle progression [24]. *WEE2* (WEE1 homolog 2) also contains predicted ZAR1 TCS (Additional file 1: Figure S6a). The binding of ZAR1 to *WEE2* could not be detected due to lack of expression in cancer cells. The *WEE2* overall expression in normal tissues is low and almost limited to oocyte/testis (R2 Normal Tissues GTeX, data not shown). WEE2 is a key oocyte-specific kinase involved in the control of meiotic arrest in mice, but WEE2 has not been associated with any diseases in humans [25]. This could indicate a common regulation of *WEE* by ZAR1, but occurring in distinct tissues.

### ZAR1 tumour suppressor function is zinc-finger and p53 dependent

**Fig. 3:**
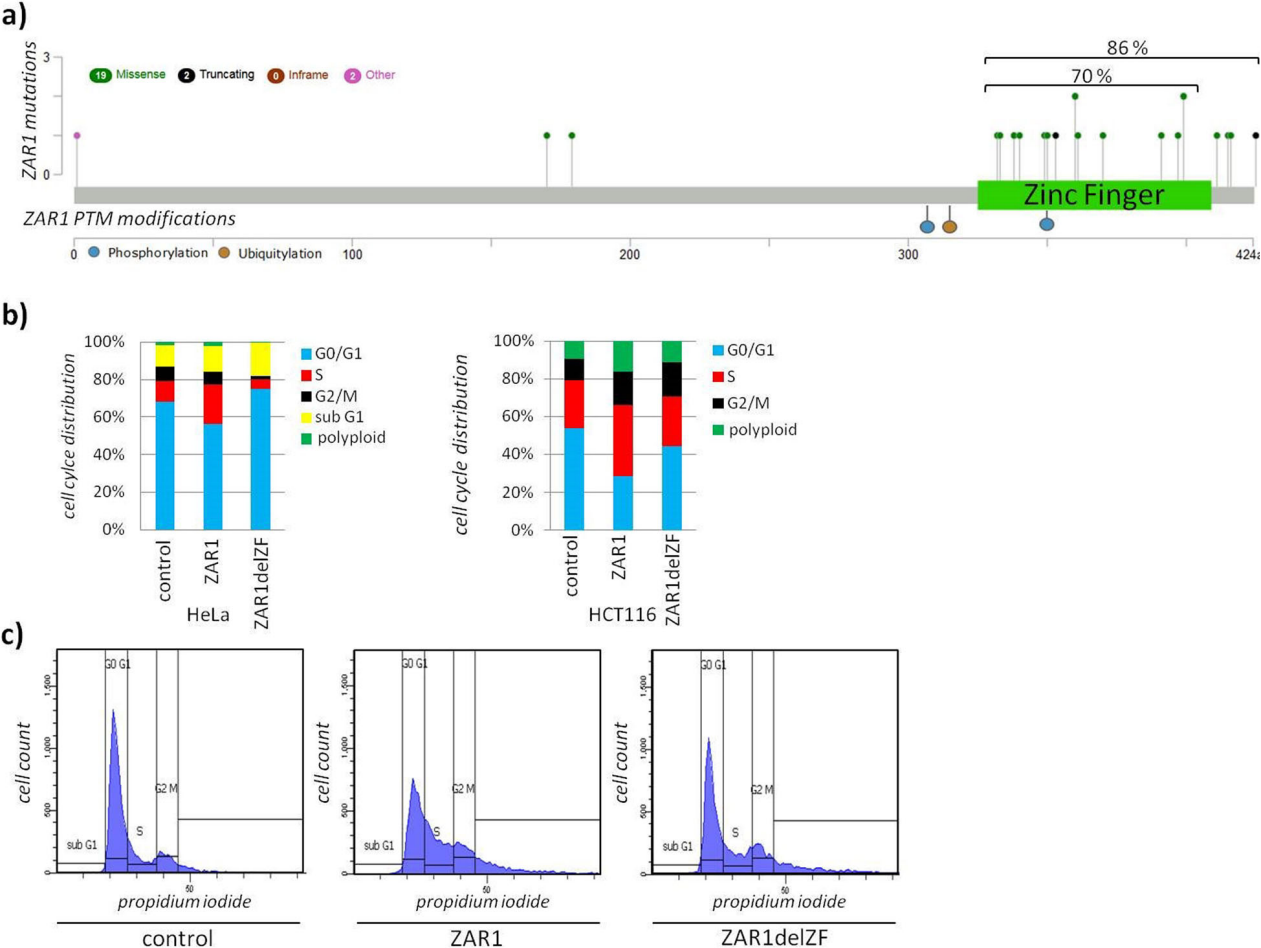
ZAR1 zinc-finger domain conveys its growth arrest potential. **a** ZAR1 Mutation mapping found in cancer patients and PTM position reveals association with zinc-finger domain (green; TCGA PanCancer Atlas patient samples; modified). **b** Quantified cell cycle arrest upon ZAR1 overexpression in HeLa and HCT116. ZAR1-EYFP, ZAR1delZF-EYFP (deleted zinc-finger), and EYFP-empty were overexpressed for 24 h and subsequently analysed by flow cytometry and propidium iodide staining. **c** Cell cycle distribution of transfected cells and propidium iodide-stained DNA content of cells with gating for cell cycle phases is exemplarily shown for HCT116 cells according to **b**)

For an in-depth view on the tumour suppressor function of ZAR1, we investigated the effects of ZAR1 reexpression in cancer. We generated transcriptomic data upon *ZAR1* reexpression/overexpression in the *ZAR1* hypermethylated cell line HCT116 (Additional file 1: Figure S2a). The cell line was used due to its known p53 status and well characterised characteristics of ZAR1 expression and methylation status. ZAR1 reexpression was found to be significantly associated with ‘G2M cell cycle checkpoint’ and ‘p53 signalling pathway’ by GSEA (enrichment plots, Fig. 2a). According and representative candidate gene expression from microarray is depicted for *WEE1*, *p27*, and *p21* upon ZAR1 reexpression (Fig. 2b). The p53 association of ZAR1 was further investigated using p53 deficient vs. wildtype HCT116 upon ZAR1 reexpression. We found that ZAR1 reexpressing cells accumulated in S phase and did not enter mitosis (Fig. 2c, d). This effect was partially p53 dependent, using p53 deficient cells. TP53 deficiency was verified earlier [26] and is shown by reduced *p21* levels (Fig. 2e). Under ZAR1 reexpression, we observed the induction of *p27* and *p21* and observed a p53 dependency (Fig. 2e upper panel). This result was cell line independent, further validating the initial observation (Fig. 2e lower panel). Next, we aimed to understand if the ZAR1 zinc-finger is involved in its tumour suppression and investigated the ZAR1 coding region. Mutation of ZAR1 is a non-frequent event in cancer patients across cancer types with an incidence of <2% (*n* = 4440 TCGA tumours, 15 cancer types, analysed using [27]). ZAR1 mutated cancers were colorectal, endometrial, lung, glioblastoma, and mamma carcinoma (analysed using [27]). Most interestingly however, mutation mapping revealed almost exclusive mutations in the C terminus/zinc-finger (Fig. 3a; 70% zinc-finger, 86% C terminus), mostly by missense mutations. The highly conserved C terminus also harbours the predicted PTM sites (Additional file 1: Figures S5 and S3a). We predicted the possible phosphorylation sites across ZAR1 by kinase analysis using *NetPhos3.1* [28] and found that position S307 may be subject to phosphorylation by p38MAPK (mitogen-activated protein kinase) and GSK3 (glycogen synthase kinase) and T350 by PKC (protein kinase C). Likewise, in mouse Zar1 p38MAPK is predicted (S244, equiv. S307 human), as is GSK3 (just slightly under threshold) and PKC (T287, equiv. T350 human). These data are consistent with a relative functional conservation of ZAR1 in vertebrates. Even in xenopus, position T221, corresponding to T350 in human, is a predicted target for PKC. Next, we generated a zinc-finger deleted ZAR1 (ZAR1delZF). We observed that ZAR1 zinc-finger deletion impaired its function, irrespective of the cell lines used (Fig. 3b). It should be taken into account that deletion of this domain could change the 3D conformation of ZAR1 and might not reflect the loss of the zinc-finger domain itself. The deletion of the zinc-finger domain also no longer arrests cell cycle progression (Fig. 3b, c); its localisation was altered (Additional file 1: Figure S9a, b) and we observed cellular stress by malformation of the nuclei, which was also partially zinc-finger dependent (Additional file 1: Figure S9c, d).

### ZAR1 is associated with ribosomal/mRNA networks, zinc-finger dependent

**Fig. 4:**
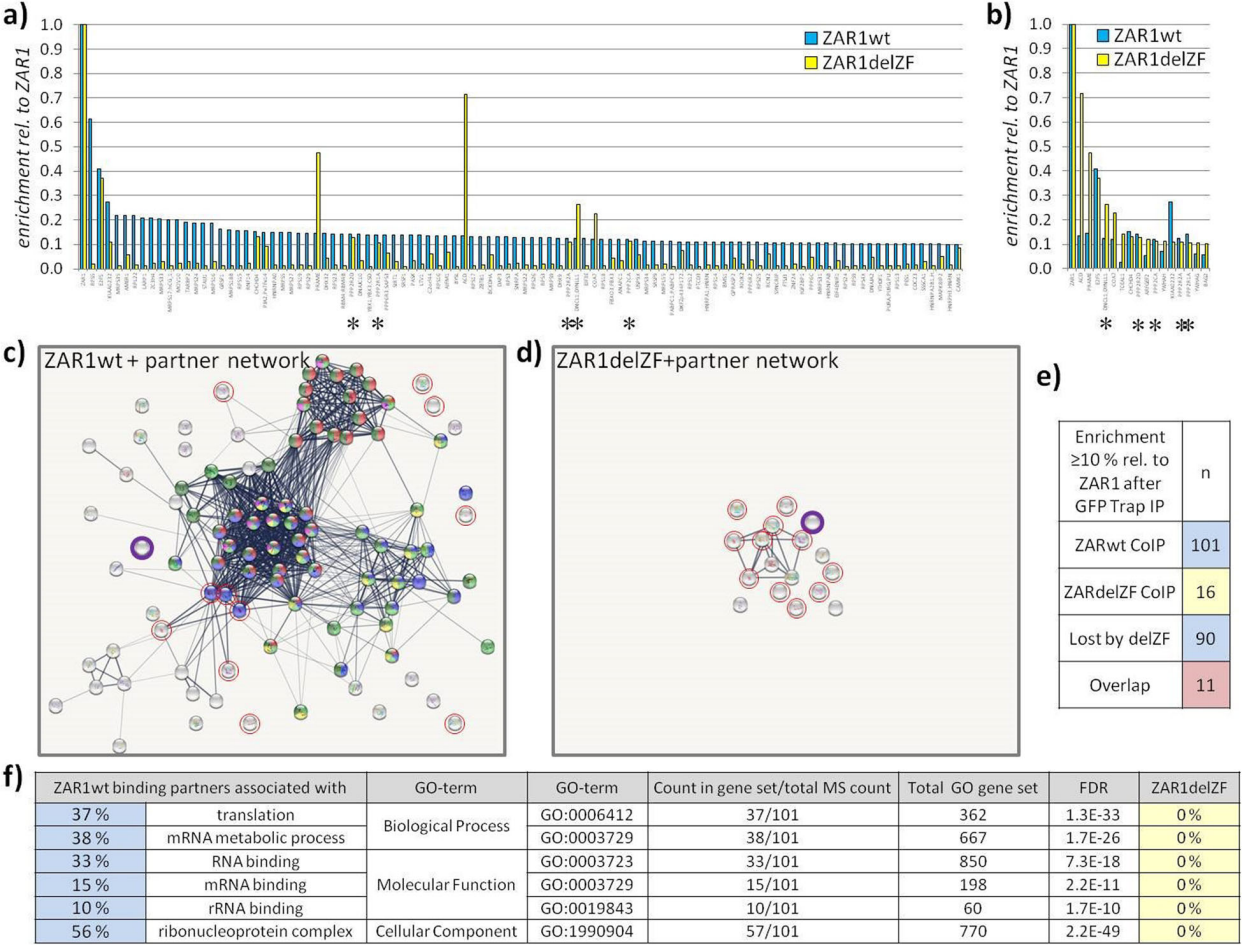
ZAR1 binding partners associate with known networks, zinc-finger dependent. **a** ZAR1wt binding partners (*n* = 101 in blue; compared to IP from ZAR1delZF in yellow) and **b** ZAR1delZF-binding partners (*n* = 16 in yellow, compared to IP from ZAR1wt in blue) from GFP Trap, and subsequent mass spectrometry with ≥10% relative enrichment to respective ZAR1 in comparison to **a** ZAR1delZF and **b** ZAR1wt. Asterisks indicate the only partner protein network-associated proteins that are unaffected by zinc-finger deletion. **c** Depiction of ZAR1wt binding partner network and **d** loss of network upon zinc-finger deletion. Red circles indicate overlap of ZAR1wt/ZAR1delZF partner proteins; ZAR1 in purple. **e** Summary of ZAR1wt and ZAR1delZF relative numbers of identified binding proteins. **f** GO-term analysis of ZAR1wt binding partners (*n* = 101) with significant enrichment. Regarding ZAR1delZF partners the GO-terms were not enriched (*n* = 16)

Neither *Pubmed*, *GPS prot* [29] nor *string* [30] searches produced known protein interaction partners or networks for ZAR1. To understand ZAR1's protein interaction network, beyond an observed zinc-finger-dependent dimer formation by co-immunoprecipitation (Additional file 1: Figure S9e), we performed ZAR1 pulldown assays followed by quantitative mass spectrometry to identify its binding partners (Fig. 4). ZAR1 binds strongly to proteins associated with known networks as defined by Szklarczyk et al. [30] (84% of ZAR1 partner proteins, Fig. 4a, c), and the strongest partner directly came from the network centre (Fig. 4a, Additional file 1: Figure S10). These identified ZAR1 partners significantly associated with the following network/GO-terms: 37% ‘translation’, 38% ‘mRNA metabolic process’, 33% ‘RNA binding’, and 56% ‘ribonucleoprotein complex’ (Fig. 4f, Additional file 1: Figure S10). In contrast, zinc-finger-deleted ZAR1 lost this strong network association (Fig. 4d). None of the remaining interacting proteins from ZAR1delZF was associated with the enriched GO-terms for ZAR1wt (Fig. 4f). Only five network-associated proteins (asterisks) are binding to ZAR1 zinc-finger independently (Fig. 4a, b, Additional file 1: Figures S10 and S11), which were not amongst the strongest ZAR1 partners (Fig. 4a). Deletion of the zinc-finger from ZAR1 lost 90 interacting proteins from ZAR1wt (total *n* = 101, Fig. 4e). There was a remaining overlap of 11 proteins with 5 being present in the ZARwt network. Interestingly, the ZAR1delZF-remaining partner proteins associated weakly with ‘mitotic cell cycle phase transition’- and ‘dephosphorylation’-related proteins (Additional file 1: Figure S11). In conclusion, we found that ZAR1 network interaction strongly depended on its C-terminal zinc-finger domain.

### Epigenetic therapy reactivates the ZAR1 tumour suppressor

**Fig. 5:**
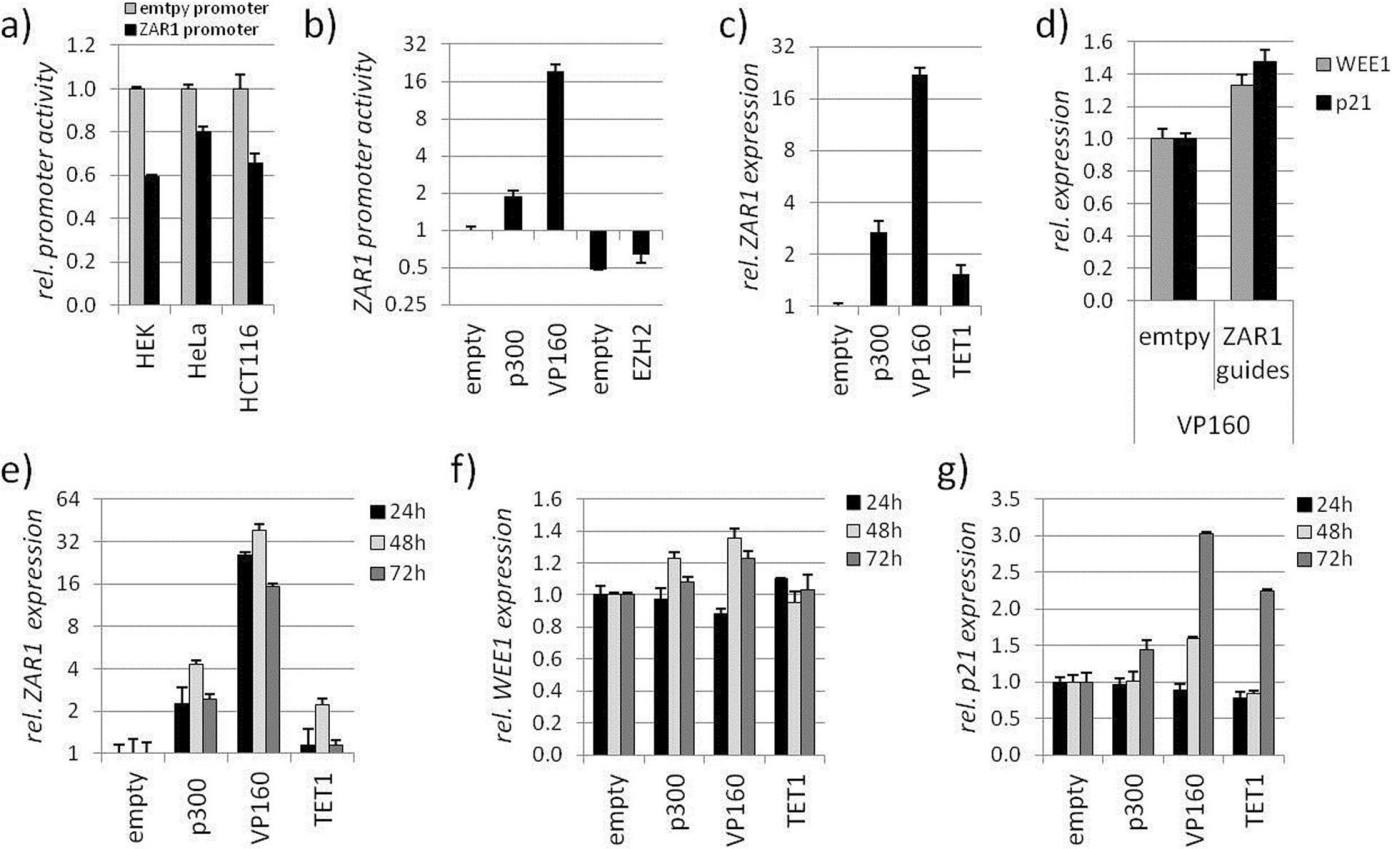
Epigenetic therapy of *ZAR1* induces ZAR1 targets *p21* and *WEE1*. **a** ZAR1 promoter activity is inhibited in comparison to pRLnull empty reporter construct by luciferase assay. **b** ZAR1 promoter activity can be epigenetically modified by the overexpression of guide RNAs targeting ZAR1 together with epigenetic activators p300, VP160, and epigenetic inhibitors EZH2 and DNMT3A by luciferase assay. **c** *ZAR1* endogenous expression in HeLa can be reactivated by epigenetic activators p300, VP160, and TET1 targeted to ZAR1 by RNA guides by RT-PCR. **d** Expression of ZAR1 targets *WEE1/p21* by RT-PCR is stimulated by overexpression of ZAR1 guided VP160. **e, f, g** Epigenetic reexpression of *ZAR1* by overexpression of ZAR1 guide directed p300, VP16,0 and TET1 or empty control for 24, 48, and 72 h as well as expression dynamics of reexpressed ZAR1 targets *WEE1* and *p21* by ZAR1-guided epigenetic activators

**Fig. 6:**
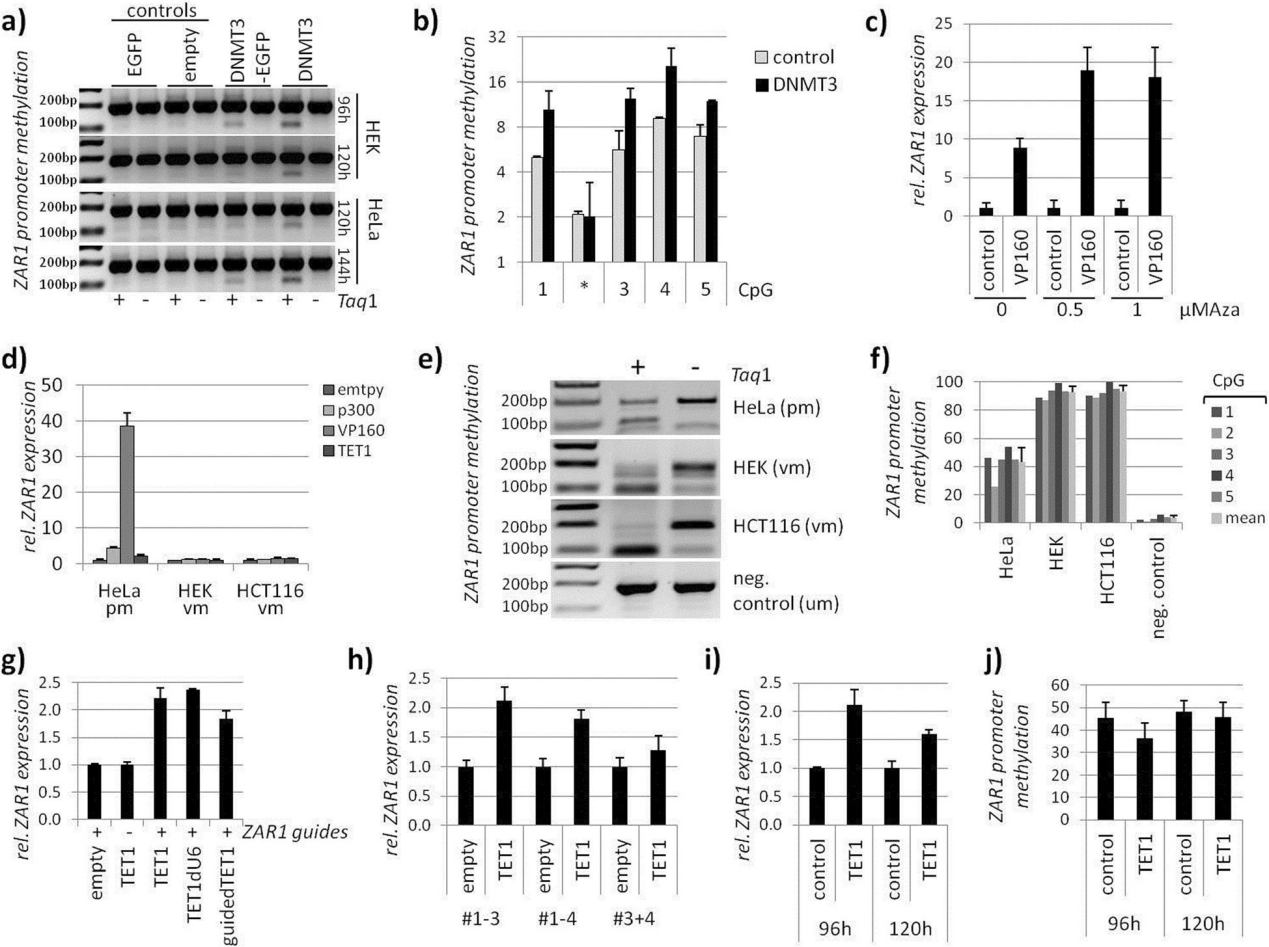
Modulation of ZAR1 promoter methylation offers therapeutic approach to ZAR1 reactivation. **a** Hypermethylation of ZAR1 by DNMT3A using ZAR1 promoter (pRLnull) in HEK and HeLa for indicated time points followed by CoBRA methylation analysis and **b** according quantification by pyrosequencing. **c** Pharmacological DNMT inhibition by 0.5 μM/1 μM 5-aza-2′-deoxycytidine (Aza) together with ZAR1 targeted VP160 overexpression activates *ZAR1* expression. **d** *ZAR1* endogenous reexpression by epigenetic editing through overexpression of ZAR1 guided p300, VP160, and TET1 in HeLa, HEK, and HCT116. **e** Promoter methylation of HeLa, HEK, and HCT116 by CoBRA and **f** quantified by pyrosequencing. **g** *ZAR1* endogenous reexpression by epigenetic editing through ZAR1-guided TET1 upon overexpression **h** is guide-combination dependent. **i, j** *ZAR1* reexpression by overexpressed TET1 (guided by ZAR1 oligos) is accompanied by ZAR1 demethylation by pyrosequencing

At last, we aimed for a therapeutic and targeted epigenetic approach to reactivate the ZAR1 tumour suppressor in cancer. We used CRISPR-dCas9 (nuclease deficient) fused to epigenetic modulators [31]. We used the modulators for DNA methylation: DNMT3A, which is a DNA methyltransferase [32] and TET1, which is a methylcytosine dioxygenase [33]. We further used modulators for histones: p300, which is ahistone acetyltransferase [34] and EZH2, which is a histone-lysine methyltransferase [35]. The VP160 activator [36] is a CRISPR transcriptional activator derived from herpes virus protein VP16 [37]. These activators and inactivators were chosen to show the transferability of epigenetic therapy to several modes of action at the ZAR1 promoter and associated ZAR1 expression. We designed ZAR1 guide RNAs that are covering the full ZAR1 CGI (Additional file 1: Figures S1 and S12). These RNA guides effectively target *ZAR1* genomically (Additional file 1: Figure S12). The *ZAR1* promoter itself is in a repressed state (Fig. 5a), which indicates a negative cellular control. The ZAR1 promoter, however, can be modulated by overexpressing ZAR1 guides together with modifiers (Fig. 5b). As expected, p300 and VP160 activated the *ZAR1* promoter. EZH2 and DNMT3A repressed it. These results proved to be also transferable to the endogenous *ZAR1* promoter, and *ZAR1* expression was activated by guided p300, VP160, and TET1 (Fig. 5c). HeLa cells, which are partially methylated (Fig. 6e, f), express *ZAR1* at a basal level. Therefore, further inhibition by DNMT3A or EZH2 was not feasible and was not tested. The success of the epigenetic therapy strongly depended on the amount of guides and modifiers as well as the combination and positioning of guides. We determined the optimal dose for the ZAR1 guides not only by promoter assays but also on endogenous *ZAR1* (Additional file 1: Figure S13a, c). Furthermore, we determined the optimal amount of epigenetic modifier using VP160 (Additional file 1: Figure S13b, d). We show that the presence of ZAR1 guides are necessary for the strong *ZAR1* induction by VP160, targeting VP160 specifically to *ZAR1* (Additional file 1: Figure S13e). Our initial result showed that VP160 together with ZAR1 guides #1-4 was more effective than #1-6 (Additional file 1: Figure S13f). This indicated that the RNA guides upstream of the ZAR1 translational start site are more effective. Detailed analysis of ZAR1 guides revealed that guides are most efficient when placed in an evenly distributed 400 bp frame upstream of the ZAR1-TSS (#1-3) using VP160 (Additional file 1: Figure S13g,h,i). This successful targeting and reactivation of *ZAR1* was shown on its promoter (Additional file 1: Figure S13h). These results are also nicely mirrored by likewise reactivated levels of endogenous *ZAR1* (Additional file 1: Figure S13i). Similar results were obtained for the epigenetic modifiers EZH2, DNMT3A (#1-4), and p300 (#1-3; Additional file 1: Figure S13 j,k,l). Upon optimization, we were able to reactivate the tumour suppressor *ZAR1* endogenously 20 to 45-fold using guided VP160 (Fig. 5), which in turn induced the ZAR1 target genes *p21* and *WEE1* (Fig. 5d). These levels were comparable to the ZAR1 overexpression effects on *p21* and *WEE1* (Fig. 2). Interestingly, effects of reactivated ZAR1 on *WEE1* and *p21* followed different dynamics. The *ZAR1* reactivation was also not limited to VP160, but also observed for p300 and TET1 (Fig. 5e, f). Reactivation of ZAR1 was strongest after 48 h overexpression of p300, VP160, and TET1 (Fig. 5e). *WEE1* activation was observed for p300 and VP160-driven ZAR1 activation after 48 h. This finding was less prominent after 72 h (Fig. 5f). The ZAR1-driven *p21* induction was strongest by p300, VP160, and TET1 after 72 h (Fig. 5g). This activation was already present for VP160 after 48 h. Additionally, we found cell cycle alterations upon VP160-driven ZAR1 reactivation with a slight increase of G1 phase (68% to 70%), an increase of S phase (18% to 19%), and a reduction of G2/M (14% to 12 %). These findings are in line with the observed ZAR1 overexpression-induced cell cycle arrest (Figs. 2 and 3). We published earlier that pharmacological inhibition of DNMTs using Aza (5-aza-2′-deoxycytidine) reactivated *ZAR1* expression and reduced *ZAR1* methylation [5]. Using reduced Aza amounts [5] not activating *ZAR1* alone, we recovered *ZAR1* reactivation when combined with targeted VP160 (Fig. 6c). This prompted us to investigate the direct interference with the *ZAR1* DNA methylation status to achieve its reactivation. Two ZAR1 guided DNMT3A vectors induced methylation of the artificial *ZAR1* promoter (Fig. 6a, b). We next compared fully methylated (HEK and HCT116) and partially methylated cell lines (HeLa, Fig. 6e, f quantification). We observed that partial methylation of the *ZAR1* promoter allowed its epigenetic modulation by p300, VP160, and TET1 (Fig. 6d) as seen for HeLa cells. The full methylation of the *ZAR1* promoter did not respond to epigenetic modulation. Finally, we tested TET1 to demethylate the *ZAR1* promoter. This would prove to us the therapeutic utility of epigenetic *ZAR1* modification by the CRISPR-dCas system. The TETs demethylate DNA by an indirect mechanism of converting the methylated cytosins further ultimately reaching unmethylated cytosin. TET1 demethylates DNA by oxidation of 5mC (5-methylcytosins) to 5hmC (5-hydroxyl methylcytosine) as the initial step of active DNA demethylation in mammals [38]. TET1 was guided either by separate administration of ZAR1 guides (TET1, TET1dU6) or by guides directly cloned into the TET1 vector (guidedTET1). Guiding TET1 to the *ZAR1* promoter successfully reactivated *ZAR1* expression (Fig. 6g) and was strongest when the guides were placed upstream of the TSS (#1-3, Fig. 6h). We show here that guided TET1 successfully decreases methylation levels of the *ZAR1* promoter (Fig. 6j), which is accompanied by the reexpression of *ZAR1* (Fig. 6i). The mode of action at the *ZAR1* promoter is summarized and depicted in Additional file 1: Figure S14.

In summary, we report here that *ZAR1* is epigenetically inactivated across cancers and has prognostic value for lung and kidney cancer as a biomarker. We show evidence for its tumour suppressor function, depending on its zinc-finger domain and its mRNA binding ability. Using the CRISPR-dCas9 tools, we were able to prove that epigenetic editing and reactivation of the *ZAR1* tumour suppressor is feasible.

## Discussion

ZAR1 came to our attention due to a 450k methylation array in which we identified it as one of the most strongly methylated target genes in a lung cancer cell line [5]. Since then we were curious to understand its epigenetic inactivation in cancer and its role in carcinogenesis. *ZAR1* is not only strongly hypermethylated across various cancers types but also across its complete CGI. We also show that *ZAR1* methylation is a suitable biomarker for lung and kidney cancer. Our results clarify the in part contradicting earlier reports on *ZAR1* methylation. In cancer, *ZAR1* is under an epigenetic control, which is a common theme for tumour suppressors in carcinogenesis [39]. We did not observe frequent mutation events in ZAR1 and conclude that hypermethylation is the dominant inactivating mechanism in cancer. Using epigenetic editing, we were able to modulate ZAR1 expression and methylation in cancer cell lines, further proving its epigenetic inactivation mechanism. Using TET1 as an epigenetic modifier, we could even show the demethylation of the *ZAR1* promoter that reactivated *ZAR1* expression. The use of epigenetic therapy in the reactivation of tumour suppressors has been discussed recently [40, 41]. We believe that our findings are suggesting ZAR1 reactivation in cancer as a promising target to such intervention.

With the present study, we demonstrated tumour suppressor properties of ZAR1, which were dependent on its zinc-finger domain. We also showed that ZAR1 is an mRNA binding protein and ZAR1 associates with the mRNA/ribosomal/translational network, depending on its zinc-finger. We hypothesise that ZAR1 binds its mRNA targets and thereby regulates their translation. In the future, we intend to explore the role of ZAR1's zinc-finger-dependent dimerization and post-translational regulation in its mRNA binding ability, as well as the mRNA-binding-dependent interactome. Our kinase predictions based on sequence references are hinting towards phosphorylation of ZAR1, which may regulate its stability or interaction with partners. Further studies will reveal if and how ZAR1 is controlled post translation. We believe that our work proves that, beyond its initially reported growth-controlling role in the oocyte [6], ZAR1 has an exciting additional role in tissues, where it controls cellular growth and contributes to cancer suppression. *ZAR1* promoter hypermethylation and subsequent epigenetic inactivation of *ZAR1* on the other hand contributes to carcinogenesis.

We have discovered ZAR1 as a potential cancer biomarker, which should be followed by assay development and analytical validation, clinical utility validation, and ultimately clinical implementation [42]. We suggest that determining the *ZAR1* methylation levels could serve as a convenient biomarker in the future. The advantages of DNA as a biomarker is its superior stability in cells and in body fluids, where free circulating DNA is present [43]. Methylation of DNA is a covalent bond, stable and well detectable by the bisulfite conversion method [44]. Bisulfite treatment of DNA, the gold standard for DNA methylation analysis [44–46], high-throughput bisulfite conversion [47] as well as digital droplet PCR (amplification of low levels of DNA in disproportionate sample/target combinations) [48] are available. DNA samples may be taken from tumour resections, biopsies, or from liquid biopsy material as a non-invasive method [49]. Circulating tumour cells or circulating tumour DNA are present in blood, body fluids, or even in exhaled breath condensates [50]. The latter are in clinical trials [51, 52], and liquid biopsies are FDA approved (lung cancer EGFR mutation tests as companion diagnostic) [53]. We believe that in the future, also *ZAR1* methylation has the potential to be part of cancer screens. The FDA has already approved of several cancer biomarkers that are in clinical practice for, e.g., liver, prostate, ovarian, breast, pancreatic, lung, and thyroid cancer [54, 55], and there is a DNA methylation marker screening available for colorectal cancer, which is blood based [56]. In our study, we show that epigenetic therapy of *ZAR1* is achievable. In the future, we are anticipating that targeted therapies will also include epigenetically inactivated tumour suppressors by, e.g., the CRISPR-dCas9 technique and viral application of epigenetic editors to reactivate not only ZAR1 *in vivo* in cancer.

## Conclusion

For the first time, our study presents evidence that ZAR1, which harbours tumour suppressive properties, is a prognostic and diagnostic cancer biomarker. ZAR1 suppresses tumour cell line growth in part through p53 and strongly depending on its functional zinc-finger. Ultimately, we found that ZAR1 can be reactivated by epigenetic therapy using the CRISPR dCas9 system.

## Methods

### Methylation analysis. CpG Island prediction, PCR product size, and digestion products

The promoter region of *ZAR1* was analyzed by CpG plot http://www.ebi.ac.uk/Tools/seqstats/emboss_cpgplot/ and *UCSC genome browser*. Primers for bisulfite-treated DNA were designed to bind only fully converted DNA and amplify promoter region. The precise promoter region was chosen for CpG content and presence of according restriction enzymes for CoBRA analysis. The size of the *ZAR1* CoBRA PCR product is 186 bp (with *Taq*I site at 89). For further details on CoBRA analysis see Richter et al.'s study [57].

### DNA Isolation, CoBRA, and Pyrosequencing

DNA was isolated after proteinase K (Thermo Fisher Scientific) digest and extracted either with phenol/chloroform or by QIAamp DNA extraction kit (Qiagen), and concentrations were determined. For CoBRA methylation analysis, a total of 2 μg genomic DNA was bisulfite treated (5 mM hydroquinone, 1.65 M sodium metabisulfite, and pH 5.5 with 0.025 M NaOH) and incubated overnight at 50 °C. DNA was purified using MSB Spin PCRapace (STRATEC Molecular), eluted in 50 μl H_2_O, and followed by 10 min incubation with 5 μl 3 M NaOH at 37 °C. DNA was then precipitated with 100% ethanol and ammonium acetate and resolved in 1 × TE buffer. Alternatively, we used 500 ng genomic/plasmid DNA and the EZ DNA methylation kit (Zymo research) according to manufacturer's protocol. Bisulfite DNA was used for CoBRA PCR. The subsequent PCR product (CoBRA primers) was digested with 0.5 μl of *Taq*I (Thermo Fisher Scientific) 1 h at 65 °C and resolved on 2% TBE gel (×0.5) together with mock control and DNA ladder. Pyrosequencing (incl. five CpGs) was performed according to manufacturer's protocol with PyroMark Q24 System (Qiagen). When analysing methylation of the artificial ZAR1 promoter by pyrosequencing the 2, CpG was not present due to mutation of the cloned ZAR1 promoter. This allowed us to distinguish between genomic and ZAR1pRLnull plasmid being isolated and pyrosequenced. *In vitro*, methylation (pos. control) of genomic DNA was performed using CpG Methyltransferase *M.Sss*I (NEB) according to manufacturer's protocol.

### RNA expression analysis

RNA was isolated from human cell culture using Isol-RNA lysis procedure (Trizol, Thermo Fisher Scientific). RNA was *DNase*I (Thermo Fisher Scientific) treated and then reversely transcribed by MMLV (Promega). Quantitative RT–PCR was performed in triplicate with SYBR select (Thermo Fisher Scientific) using Rotor-Gene 3000 (Qiagen) and normalised to *GAPDH/ACTB*. We performed RNA microarrays (Clariom S human) according to manufacturer's protocol (P/N 703174 Rev. 2) with 200 ng of total RNA. Reagents/equipment were GeneChip WT PLUS Reagent Kit, P/N: 902280; GeneChip Hybridization, Wash, and Stain Kit P/N 900720, GeneChip Scanner, GeneChip Fluidics Station 450, GeneChip Hybridization Oven 640, Bioanalyzer 2100 (Agilent), and RNA600 NanoKit (Agilent).

### Identification of RNA binding using crosslinking and immunoprecipitation (CLIP)

Potential binding of Zar1 to RNA in general and specifically to the WEE1 mRNA was tested using a shortened version of the CLIP procedure [58]. HEK293T cells were transfected with ZAR1-EYFP or EYFP-empty for 30 h and UV irradiated (254 nm; 300 mJ/cm^2^) to crosslink RNA binding proteins to cellular RNA. After cell lysis, the RNA is trimmed by limited *RNase*I digestion. ZAR1 is immunoprecipitated by GFP Trap (ChromoTek). The 2′,3′-cyclic phosphate produced by *RNase*I digestion is removed by phosphatase treatment. The RNA is radioactively 5′ end-labelled with ^32^P. Free RNA is removed by gel electrophoresis followed by transfer to a nitrocellulose membrane, which binds proteins unspecifically. After autoradiography, the area with the covalent protein/RNA complexes of interest is cut from the membrane. The RNA is eluted from the membrane by protein digestion with proteinase K. RNA is reversely transcribed before RT-PCR.

### Cell lines, lung cancer tissues, and controls

Cell lines used were published earlier. FTC-133 [59], Hep2 [60], Hep2G [61], A549 [62], HCT116wt, and HCT116delp53 were obtained from Thorsten Stiewe [63]. RD [64, 65], germ cell carcinoma [66], malignant melanoma cell lines [67], kidney cancer cell lines [60], breast cancer cell lines [57], glioblastoma cell lines were obtained from Lienhard Schmitz [68], ovarian carcinoma samples, and control patient material; and all patients signed informed consent before enrolment [69]. The study was approved by local ethic committees [69].

### Cell culture, cell cycle analysis, and ZAR1 localisation

Cell lines were grown in appropriate medium (DMEM or RPMI) supplemented with 10% FCS and 1% Penicillin/Streptomycin under cell culture conditions (37 °C, 5% CO_2_). Cell lines were transfected using Turbofect (Thermo Fisher Scientific), X-tremeGENE HP (Roche), or Polyethylenimin (Sigma) with either 4 μg (6 wells) or 10 μg (10-cm dishes). Regarding flow cytometry analysis, cells were transfected and ethanol fixed at indicated time points. The following day, cells were treated with 50 μg/ml *RNase*A for 30 min at 37 °C. Subsequently, cells were stained with 50 μg/ml propidium iodide prior to measuring DNA content in FACSCantoII (BD Biosciences). FACSDiva Software (BD Biosciences) was used for measurement/gating to distinguish transfected fluorescent cells and to determine cells in G0/G1, S, and G2/M phases of the cell cycle. For localisation analysis, cells were seeded on glass slides and transfected the following day. Cells were fixed with 3.7% formaldehyde at according time points, permeabilised using tritonX, stained with DAPI (0.1 μg/ml in PBS, Sigma), embedded in anti-fading with Mowiol (Sigma), and analysed with Axio Observer Z1 (Zeiss) under ×63 magnification and Volocity Software (Perkin Elmer). Analysis of ZAR1 was restricted to overexpressed ZAR1 due to commercial antibodies not being useful for endogenous ZAR1 detection in western blotting and immunofluorescence.

### Plasmids and promoter reporter assay

*ZAR1* coding sequence was cloned into pEGFP-C2 [5] (Clontech), pEYFP (Clontech), pCMVTag1 (Flag; Agilent), and pEBG (GST+Flag). The ZAR1 zinc-finger was deleted by site-directed mutagenesis (QuikChange Lightning, Agilent) and verified by sequencing, western blotting, and fluorescence microscopy. The ZAR1 promoter (position −530 to +76 relative to transcriptional start site) was amplified from genomic DNA and cloned into pRL-null (Promega) [5]. We used the Dual-Glo-Luciferase Reporter Assay System (Promega) according to manufacturer's protocol and pGL3 for transfection/expression control. Cell lines were chosen due to determined ZAR1 status (methylation and expression) and being well established cancer cell line models. Cell lines show a superior transfection efficiency. HEK, HeLa, or HCT116 cells were transfected for 24 h with pRLnull empty or ZAR1 promoter containing pRLnull together with ZAR1 guides in px549dCas or empty-px549dCas, and together with epigenetic effectors or empty-pcDNA3.1 control and with pGL3 transfection control. Lysates were prepared after 24 h, and luciferase was measured. Renilla luciferase promoter results (pRLnull) were normalised to pGL3 (firefly luciferase). ZAR1pRLnull was normalised to pRLnull empty. Measurements were done in triplicates. Controls were set 1. Transfections were performed at 80% cell density in 6 wells with a total of 4μg Plasmid using PEI.

### Epigenetic editing/ Epigenetic therapy by CRISPR-dCas9

CRISPR-Cas9 vector px549 was obtained from Lienhard Schmitz (Giessen, Germany) and adapted for epigenetic editing by inactivation of Cas9 (dCas9 site-directed mutation). *ZAR1* guide RNAs/Oligos were positioned/generated using Benchling (Additional file 1: Figure S1a) [70] and cloned into px549-dCas and TET1 through the *Bbs*I site. Epigenetic modifier plasmids were ordered from Addgene and modified if indicated: pcDNA-dCas9-p300 Core (61357), pdCas9-DNMT3A-EGFP (71666) with deletion of U6 promoter (site-directed mutagenesis), pdCas9-Tet1-CD (83340) as wildtype, with deletion of U6 promoter (site-directed mutagenesis) or as wildtype with cloned ZAR1 guides in *Bbs*I restriction site (ZAR1-guided-TET1), pcDNA3.1-MS2-Tet1-CD (83341), Ezh2[SET]-dCas9 (100087), DNMT3A-dCas9 (100090). Epigenetic editing of endogenous ZAR1 was performed in the ZAR1 partially methylated Hela, if not mentioned otherwise. ZAR1 RNA guides are #1 ACTTTCGCTCACTTAGCCAG, #2 TGGTTCCCTTACGGATCAGC, #3 GTAGGGAGAAGGACGAAGAG, #4 GTCGCCTATTTAGGGTGCGG, #5 CGCGGCCACCAAGGGCAAGG, and #6 CCGCGGTACAGTGCTCGCTG and are positioned relative to TSS at −402 #1, −230 #2, −133 #3, −3 #4, +120 #5, and +386 #6.

### Binding partner identification using GFP Trap and mass spectrometry

ZAR1-EYFP, ZAR1delZF-EYFP vs. EYFP-empty were overexpressed in HEK293T cells (24 h), and pulldown was performed according to manufacturer's protocol by GFP Trap (ChromoTek). Triplicate sample pairs were processed by off-bead digest, strong anion exchange (SAX) extraction, and dimethyl-labelling, followed by LC-MS^2^. I brief, beads were resuspended in two volumes urea buffer (6 M urea, 2 M thiourea, 10 mM dithiothreitol, 10 mM HEPES, pH 8.0) and incubated shaking for 30 min at room temperature. Cysteins were alcylated at 55 mM final concentration of iodoacetamide, shaking at room temperature and in the dark for another 30 min. Peptidolysis was then initiated with 0.5 μg Lys-C (Wako Chemicals GmbH) for 3 h shaking at room temperature, followed by dilution to 2 M urea/thiourea, addition of 0.5 μg trypsin (Serva) and an overnight shaking incubation at room temperature. Peptide-containing supernatants were brought to 1% NH^3^ and loaded onto three-layer SAX tips equilibrated previously with 30 μl of 0.1% NH^3^. After sequential washes with 30 μl 0.1% NH^3^ and 30 μl NH^3^ in 2-propanol, respectively, columns were syringe dried, peptides eluted using 30 μl 80% acetonitrile, 0.1% formic acid and vacuum dried. In-solution chemical labelling was performed as described [71, 72]. Peptides were resolubilized and acidified using a final concentration of 0.1% TFA. Free amines were differentially modified by reductive dimethylation. The labelling reaction was quenched on ice using ammonia solution and formic acid. Differentially labelled samples were mixed 1:1 by volumes and desalted on oligo R3 columns. The subsequent LC-MS^2^ analysis used an in-house packed 70 μm ID, 15 cm reverse phase column emitter (ReproSil-Pur 120 C18-AQ, 1.9 μm, Dr. Maisch GmbH) with a buffer system comprising solvent A (5% acetonitrile, 1% formic acid) and solvent B (80% acetonitrile, 1% formic acid). Relevant instrumentation parameters are extracted using MARMoSET [73] and included in the supplementary material. Peptide/protein group identification & quantitation was performed using the MaxQuant suite of algorithms [74, 75] (v. 1.6.3.4) against the human uniprot database (canonical and isoforms; downloaded on 2019/01/23; 169,389 entries) using the parameters documented in the supplementary material.

### Further analysis of publicly accessed data and origin of data

Gene expression, promoter methylation correlation, and Kaplan–Meier calculations were performed using *R2 Genomics Analysis and Visualization Platform* [76], *Wanderer* [77]*, KM Plotter* [78–81], and *MethSurv* [82]. Gene set enrichment analysis was performed using *GSEA* [83]. The following are listed in order of appearance with resource of data. Additional file 1: Figure S1 *ZAR1* expression in human normal tissues, HPA RNA-seq normal data, Bioproject PRJEB4337, data [84]. *ZAR1* expression correlation in Cellline CCLE Cancer Cell Line Encyclopedia - Broad - 917 - MAS5.0 - u133p2, log2, ZAR1 (1555775_a_at) APS = 16.2 (407) Avg = 12.8, Source: GEO ID: gse36133 Dataset Date: 2012-03-20. Additional file 1: Figure S4 *ZAR1* expression in cancer cell lines vs. normal tissues, 1555775_a_at, log2, data Roth vs. Broad, Anova one way. *ZAR1* methylation in normal to tumour tissues and cancer cell lines, cg22773661/cg1753764, data Lokk vs. Heyn vs. Esteller, Anova one way. *ZAR1* methylation in normal to tumour tissues and cancer cell lines relative to CpG island/shores and for all *ZAR1* (cg) reporters from array. T-SNE analysis on Broad and Roth, Transform: zscore, no gene filter, no sample filter, perplexity = 50, Colour mode: Colour by Gene (*ZAR1*), Transform log 2. Overview on R2 used datasets (class,tissue,disease+additional info-author-#samples-normalisation-platform): Normal Various - Roth - 504 – MAS5.0 - u133p2, Source: GEO ID: gse7307 Dataset Date: 2007-04-09; Cellline CCLE Cancer Cell Line Encyclopedia - Broad - 917 - MAS5.0 - u133p2, Source: GEO ID: gse36133 Dataset Date: 2012-03-20; Normal Tissues - Lokk - 70 - custom - ilmnhm450, Source: GEO ID: gse50192 Dataset Date: 2014-02-26; Tumor Types (landscape) - Heyn - 493 - custom - ilmnhm450, Source: GEO ID: gse76269 Dataset Date: 2017-06-07; Cellline Cancer Pharmacogenomic - Esteller - 1028 - custom - ilmnhm450, Source: GEO ID: gse68379 Dataset Date: 2016-07-05. Figure 1: Analysis performed using *Wanderer* [77] TCGA data, gene: *ZAR1*, dataset project: TCGA, data type: 450k Methylation Array, for LUAD lung adenocarcinoma and KIRC Kidney renal clear cell carcinoma. Pan-cancer mRNA RNA-seq using *KM Plotter* [85], Tumor type: Kidney renal clear cell carcinoma and Lung adenocarcinoma, Split patients by: Auto select best cutoff, Follow up threshold: 60 months. Analysis performed using *MethSurv* [82] on TCGA cancer datasets: KIRC Kidney renal clear cell carcinoma and LUAD Lung adenocarcinoma, Relation to island: Island, Genomic Region: TSS200, Split by: mean. Additional file 1: Figure S5: Conservation and alignment of by *PhyloP; UCSC genome browser* [86] and *BioEdit* [87] matrix: BLOSUM62 on sequence ZAR1 from homo sapiens, mus musculus and xenopus laevis from *NCBI RefSeq* [88]. *Swiss-Model* [18] prediction by template ‘Pre-mRNA-splicing factor SLT11’ with sequence identity 22.22% in the range 318-380 aa and sequence similarity 0.33. PTM prediction by *PhosphoSitePlus* [27]. Figure 3: mutation analysis on TCGA PanCancer Atlas Studies through *cBioPortal* [89, 90]. Figure 4: ZAR1 binding partner Network depiction/analysis by *String v11* [30].

### Primers

Primers for CoBRA analysis of the *ZAR1* promoter (186 bp) were upper primer GGAGAAGGAYGAAGAGGGGTTTTT and lower primer TCCCCCAAAACCRCCATAAAC, and pyrosequencing primer was TGGTAGGAAGGGYGTGGAGG. Primers for RT-PCR were *ZAR1* AGCTGGGCAAGGAGCGGCTG and GGTGGGGCCGTTTAGGGTCCA (264 bp), *GAPDH* TGGAGAAGGCTGGGGCTCAT and GACCTTGGCCAGGGGTGCTA (176 bp), *ACTB* CCTTCCTTCCTGGGCATGGAGTC and CGGAGTACTTGCGCTCAGGAGGA (226 bp), *p21* CCTTGTGCCTCGGTCAGGGGAG and GGCCCTCGCGCTTCCAGGAC (183 bp), *p27* GTGCGAGAGAGGCGGTCGTG and TCCACCGGGCCGAAGAGGTT (146 bp), *WEE1* CACACGCCCAAGAGTTTGC and CACTTGAGGAGTCTGTCGCA (135 bp) and *WEE1* 3′UTR primers are: pair 1 CTCCCCCTGAACACTGTGAC and ACTGACACCAATCGAGAAAGT (87 bp), pair 2 CACCAGCCTTTCCAGGGTTA and GGTCACTACAGGGAAAGACACC (92 bp), pair 3 AGCCTTCAATGTACCTGTGTGT and TGCCTACAAAGTGCTCCCAG (93 bp), pair 4 CTGGGAGCACTTTGTAGGCA and AGCAGCAAATTCACAAGGCA (77 bp), pair 5 AGTTTTGTCTTTGCTGTAAACTTGT and CATCAAAAGCAGCTATACATTTCAC (100 bp), pair 6 TGCACCCTTTCCCTCCTTTG and GTCCGGGAAGGACATTACCA (89 bp), pair 7 TGTTTTGCCCGGTTTTTCTCT and GTCAGAAGTCATTCTGGCATTTCA (95 bp), pair 8 TTTGCACTTGTCTTTGACTTGTGT and AGGTAAGCTCAGAGTGACTTTT (70 bp), pair 9 GCCATTTGACTAATAATACTGGCT and ACACAAGTCAAAGACAAGTGC (106 bp).

## Supplementary information

**Additional file 1: Figure S1.** Overview ZAR1 genomic structure, expression pattern and GO-term correlation, **Figure S2.** *ZAR1* promoter is hypermethylated across cancer cell lines. **Figure S3.** *ZAR1* methylation in ovarian carcinoma. **Figure S4.** Epigenetic inactivation of *ZAR1* across human cancers. **Figure S5.** ZAR1 is conserved across human, mouse, and xenopus with high C-terminal homology and structure prediction. **Figure S6.** Confirming human ZAR1 RNA binding ability. **Figure S7.** ZAR1 is cytosolic. **Figure S8.** Reexpression of ZAR1 alters the transcriptome and reveals association with mRNA 3′end processing. **Figure S9.** ZAR1 function depends on its zinc-finger. **Figure S10.** ZAR1 binding partner GO-term association overview. **Figure S11.** ZAR1 binding partner GO-term association overview. **Figure S12.** Targeting the ZAR1 genomic region with CRISPR ZAR1 guide RNA oligos. **Figure S13.** Effective epigenetic editing of ZAR1 with distinct RNA guide combinations upstream the TSS.

## Data Availability

Supplementary materials are supplementary figures, data, and raw data. Microarray data are available in the ArrayExpress database (www.ebi.ac.uk/arrayexpress; through Annotare2.0) under accession number E-MTAB-8292.

## Abbreviations

CGI: CpG island
CRISPR: Clustered regularly interspaced short palindromic repeats
dCas9: deactivated Cas9
ZAR1: Zygote arrest 1
