# Supplementary material for: Epigenetic therapy of novel tumour suppressor ZAR1 and its cancer biomarker function
Source: Clin Epigenetics. 2019 Dec 4;11:182. doi: 10.1186/s13148-019-0774-2 (PMC6894338; doi:10.1186/s13148-019-0774-2)
Supplement: Supplementary file 1 — Additional file 1: Figure S1. Overview ZAR1 genomic structure, expression pattern and GO-term correlation, Figure S2. ZAR1 promoter is hypermethylated across cancer cell lines. Figure S3. ZAR1 methylation in ovarian carcinoma. Figure S4. Epigenetic inactivation of ZAR1 across human cancers. Figure S5. ZAR1 is conserved across human, mouse, and xenopus with high C-terminal homology and structure prediction. Figure S6. Confirming human ZAR1 RNA binding ability. Figure S7. ZAR1 is cytosolic. Figure S8. Reexpression of ZAR1 alters the transcriptome and reveals association with mRNA 3′end processing. Figure S9. ZAR1 function depends on its zinc-finger. Figure S10. ZAR1 binding partner GO-term association overview. Figure S11. ZAR1 binding partner GO-term association overview. Figure S12. Targeting the ZAR1 genomic region with CRISPR ZAR1 guide RNA oligos. Figure S13. Effective epigenetic editing of ZAR1 with distinct RNA guide combinations upstream the TSS. [file 13148_2019_774_MOESM1_ESM.pdf]

a)

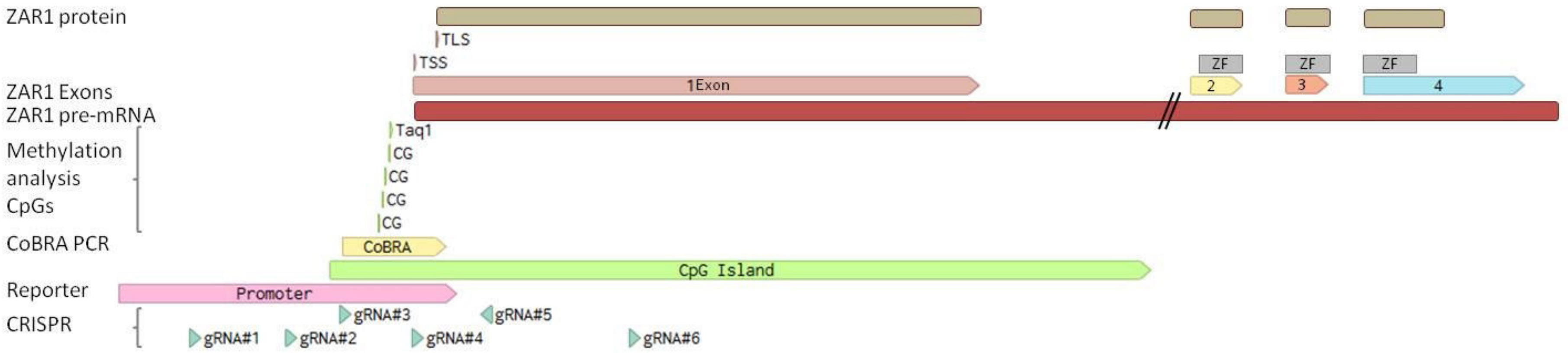

b)

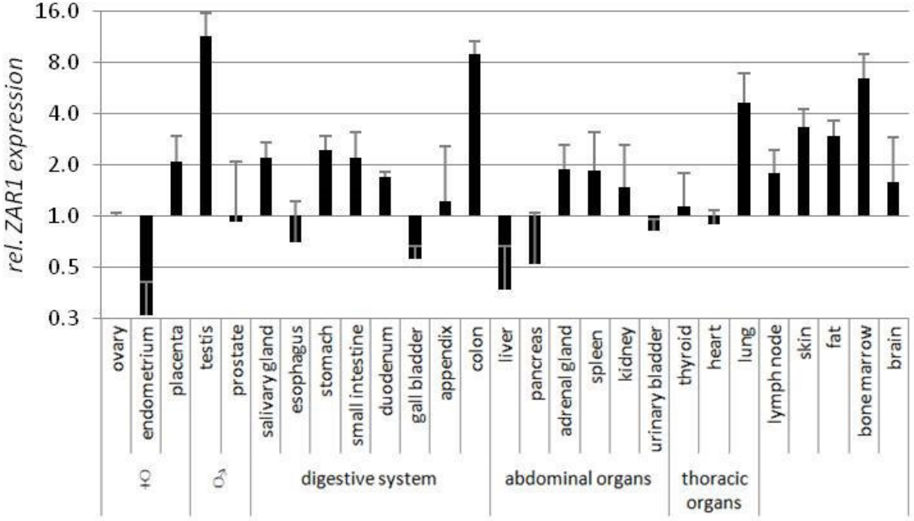

c)

GO Term Biological Process	p value	correlation	%	n			GO path
				Total	in Set	in GO	
regulation of RNA metabolic process	9.40E-04	neg.	46	26	12	3549	51252
cell communication	9.90E-06	pos.	38	210	80	5663	7154
anatomical structure development	1.30E-07	pos.	38	210	80	5196	48856
signal transduction	3.90E-03	pos.	31	210	66	5179	7165
cell-cell signaling	3.30E-10	pos.	18	210	37	1494	7267
embryonic morphogenesis	3.50E-05	pos.	7	210	15	555	48598

Suppl. Fig. 1: Overview ZAR1 genomic structure, expression pattern and GO-term correlation. a) Overview of ZAR1 structure with transcriptional start site TSS, translational start site TLS, coding sequence, zinc finger ZF, 4 exons, site for methylation analysis (CoBRA +Taq1 TCGA site, Pyrosequencing CpG), CpG island, promoter reporter construct for luciferase assay, CRISPR RNA guide localisation for epigenetic editing. **b)** ZAR1 expression is depicted across human normal tissues (relative to ovary set 1; log2; HPA RNA-seq normal data; Bioproject PRJEB4337). **c)** GO term analysis of genes associated with ZAR1 expression from Cancer Cell Line Encyclopedia reveals significant association (Expression correlation: Cellline CCLE Cancer Cell Line Encyclopedia - Broad - 917 - MAS5.0 - u133p2, log2, ZAR1 (1555775_a_at) APS=16.2(407) Avg=12.8; Source: GEO ID: gse36133 Dataset Date: 2012-03-20).

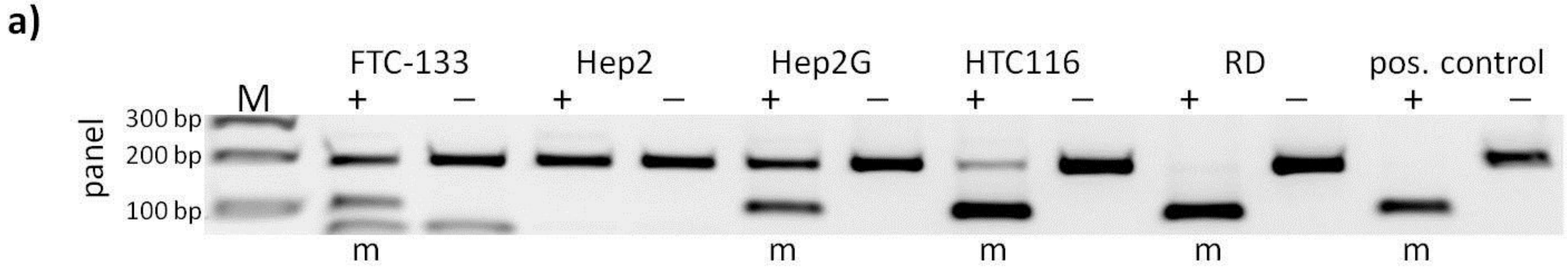

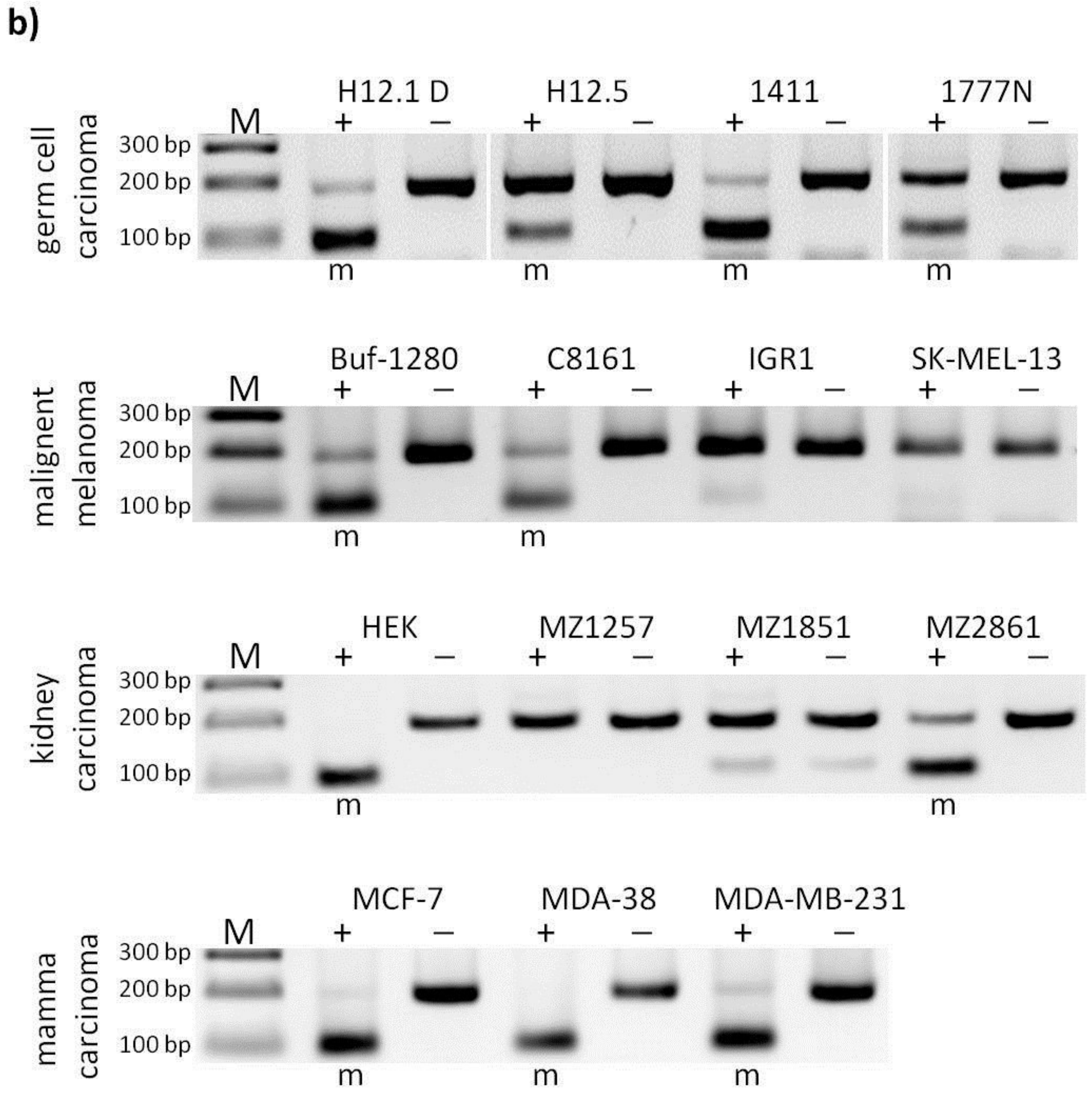

Suppl. Fig. 2: *ZAR1* promoter is hypermethylated across cancer cell lines. Methylation analysis of ZAR1 CpG island by CoBRA combined bisulfite restriction analysis for **a)** cancer cell line panel and **b)** germ cell carcinoma, malignant melanoma, kidney carcinoma, mamma carcinoma and glioblastoma cell lines. DNA was isolated, bisulfite treated, the *ZAR1* CGI promoter region was amplified by PCR, PCR products were *Taq*1 digested (+) and mock digested (-). Restriction products were separated by 2% gel electrophoresis (1xTBE; 100 bp DNA ladder, ethidium bromide stain). Methylated (m) samples are indicated by digestion products.

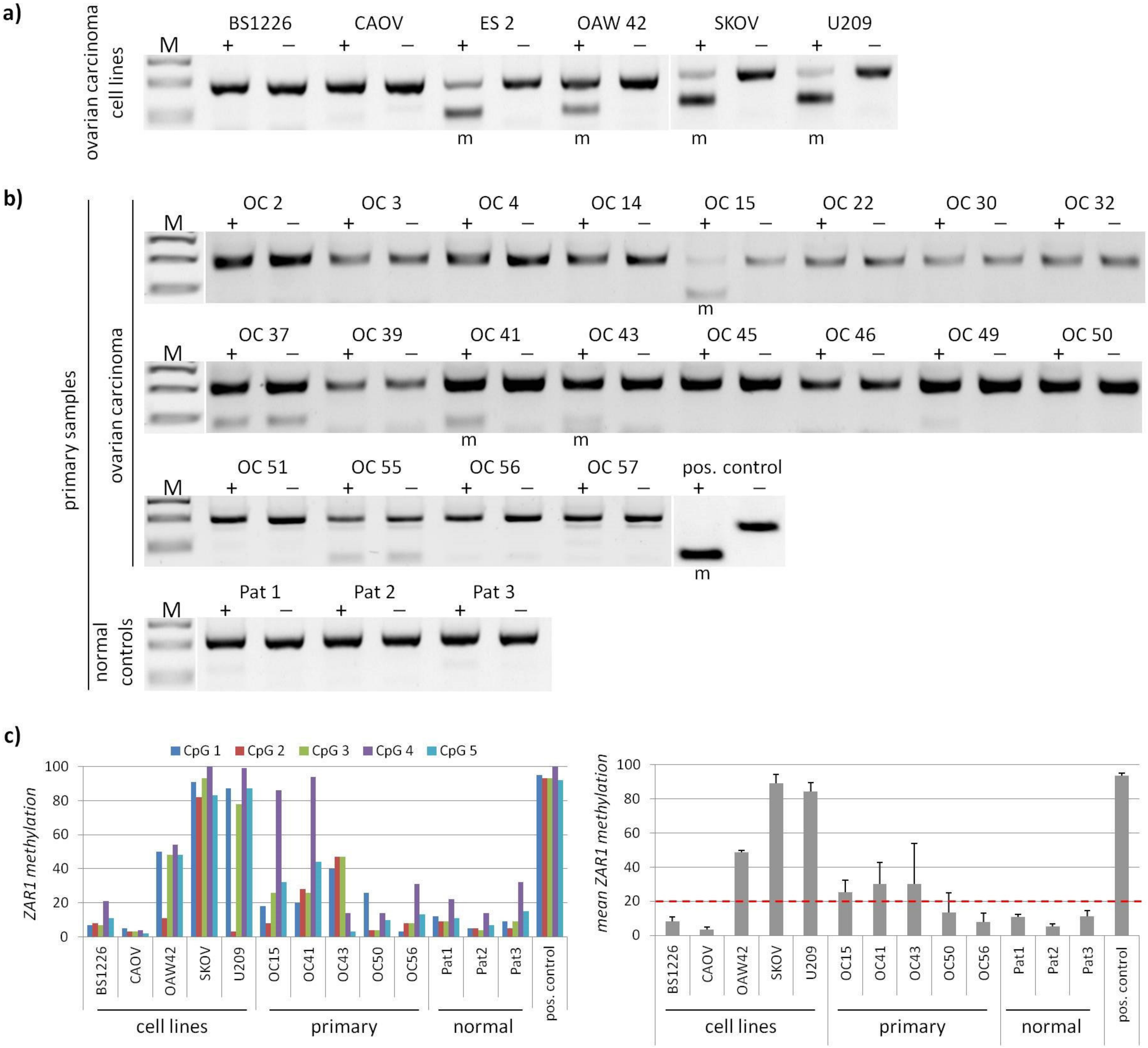

Suppl. Fig. 3: *ZAR1* methylation in ovarian carcinoma. Methylation analysis of ZAR1 CpG island by CoBRA Combined bisulfite restriction analysis for **a)** ovarian carcinoma cell line panel and **b)** primary ovarian carcinoma and control samples. DNA was isolated, bisulfite treated, the *ZAR1* CGI promoter region was amplified by PCR, PCR products were *Taq*1 digested (+) and mock digested (-). Restriction products were separated by 2% gel electrophoresis (1xTBE; 100 bp DNA ladder, ethidium bromide stain). Methylated (m) samples are is indicated by digestion products. **c)** According quantification of ZAR1 methylation by pyrosequencing is shown for five single CpGs (left) in ovary carcinoma cell lines, primary ovary carcinoma, normal control and positive control as well as the mean of 5 CpGs (right). Methylation threshold for ovarian carcinoma was defined at 20 % (red line).

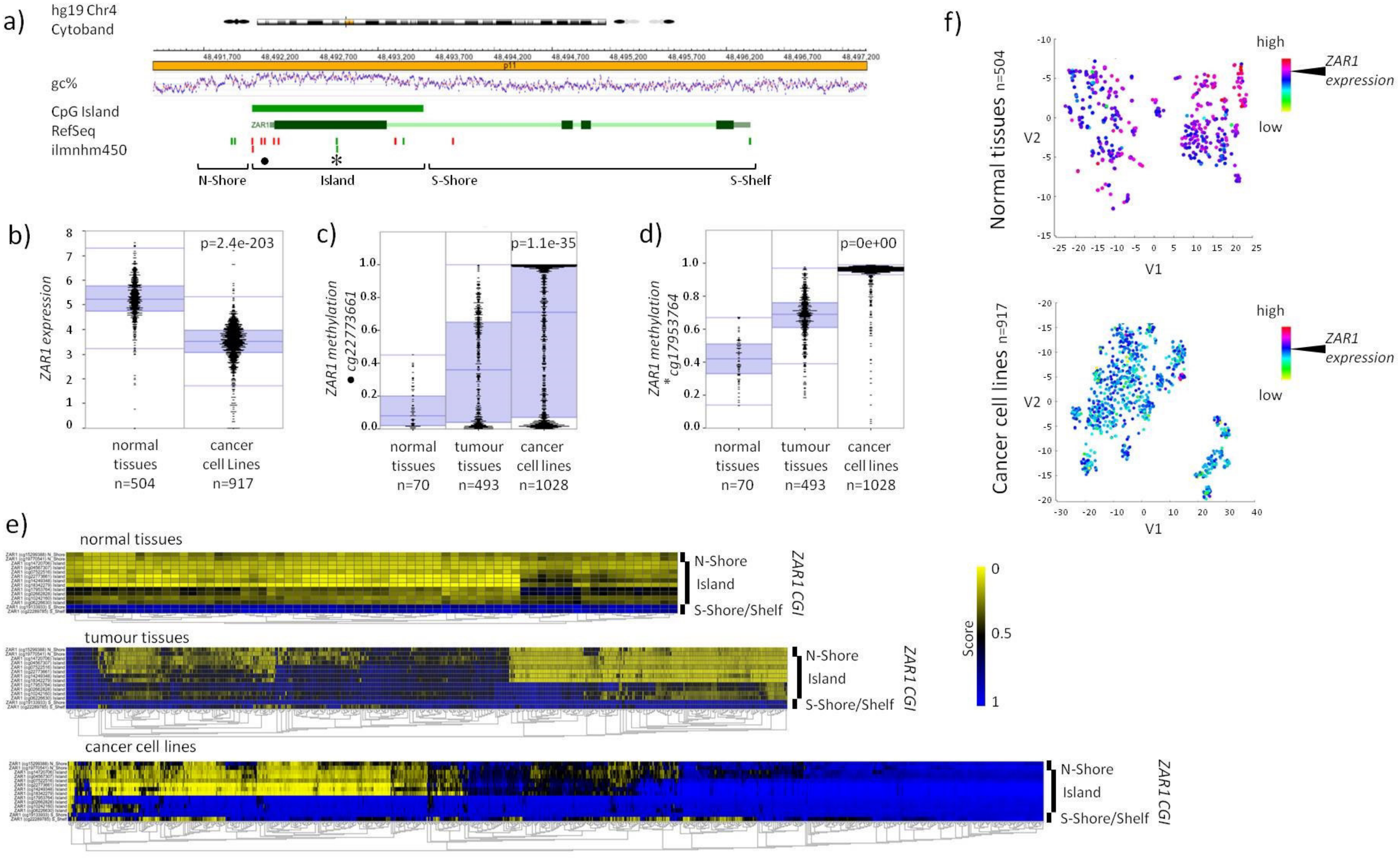

Suppl. Fig. 4: Epigenetic inactivation of *ZAR1* across human cancers. a) Genomic organization of ZAR1 with CpG Island and probe position of Illumina 450K methylation array. Asterisk/dot marks the depicted cg22773661, cg17953764 probes. **b)** ZAR1 expression is decreased in cancer cell lines vs. normal tissues (1555775_a_at, log2, data Roth vs. Broad, Anova one way). **c+d)** ZAR1 methylation increases from normal to tumour tissues and cancer cell lines (cg22773661/cg1753764, data Lokk vs. Heyn vs. Esteller, Anova one way). **e)** Increased ZAR1 promoter methylation (yellow unmethylated; blue methylated) is observed from normal to tumour tissues and cancer cell lines. Methylation is depicted relative to CpG island/shores and for all ZAR1 (cg) reporters from array. **f)** Expression comparison of ZAR1 in normal tissues (data Roth) and cancer cell lines (data Broad) by T-SNE analysis (perplexity 50; log2).

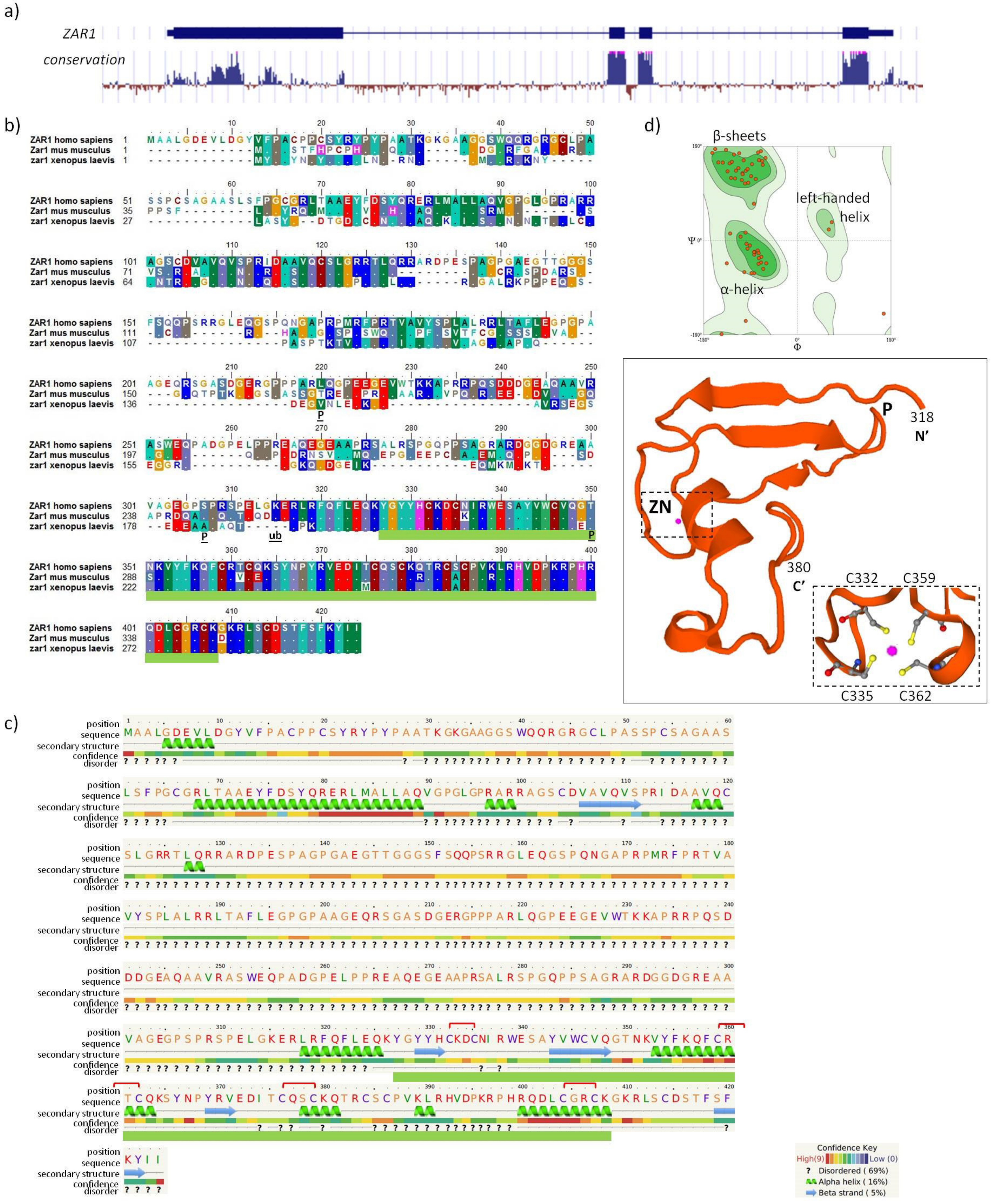

Suppl. Fig. 5: ZAR1 is conserved across human, mouse and xenopus with high C-terminal homology and structure prediction. a) ZAR1 vertebrate basewise conservation relative to *ZAR1* genomic structure *(PhyloP; UCSC genome browser)*. **b)** Amino acid sequence of ZAR1 from human, mouse and xenopus are aligned and overlap is colour coded *(BioEdit BLOSUM62)*. The ZAR1 zinc finger (human ZAR1 ZF from 327 to 408 aa) is shown as green bar and predicted phosphorylation (P) and ubiquitination sites (ub) are marked below sequence and are conserved between human and mice. **c)** Secondary structure prediction of human ZAR1 by *Phyre2* (zinc finger as green bar and 3CxxC motif red brackets). **d)** ZAR1 protein structure modelling reveals predictable zinc finger structure by Ramachandran Plot with β-sheet, α-helix and left handed helix (upper) and structure of the ZAR1 zinc finger (lower) with zinc ion (purple, ZN) and phosphorylation site is indicated (T350) using *SWISS-Model*. Zinc ion binding is shown as predicted for cysteines C332, C335, C359 and C362.

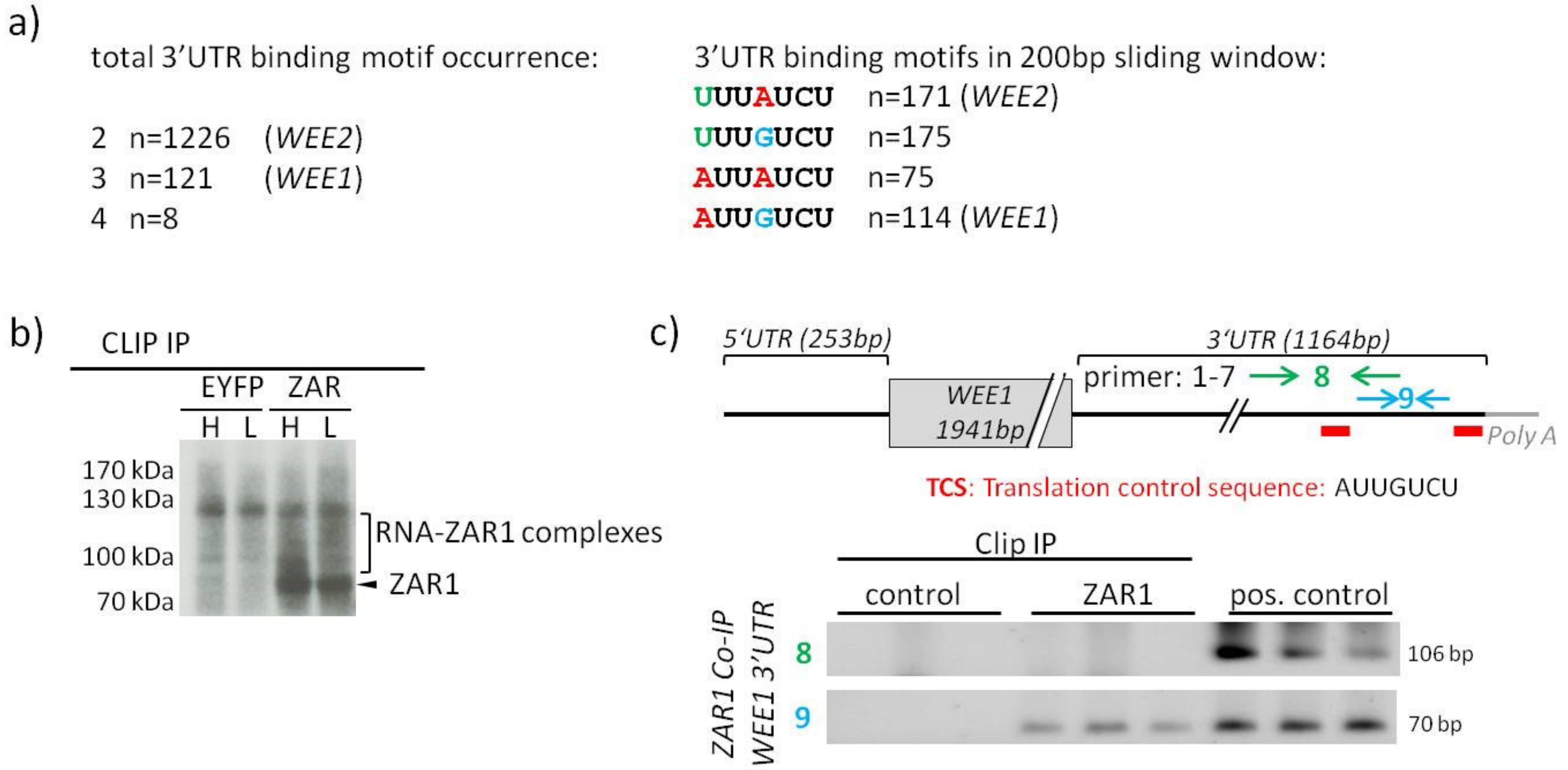

Suppl. Fig. 6: Confirming human ZAR1 RNA binding ability. a) 3'UTR binding motifs of ZAR1 in human mRNA with occurrence and duplicate motifs. **b+c) CLIP** CrossLinking and ImmunoPrecipitation: Identification of ZAR1-RNA binding by CLIP upon HEK transfection with ZAR1-EYFP and EYFP-empty for 30h. **b)** Autoradiography of CLIP with ZAR1 and ZAR1-RNA complexes under high (H 1:1000) and low (L 1:5000) *RNaseI* digest conditions. Protein marker is indicated. **c)** *WEE1* RNA structure depicts its UTRs, coding region, TCS (red) and primer pairs used to detect ZAR1 binding to *WEE1* 3'UTR. Verification of 3'UTR *WEE1* binding by ZAR1 in CLIP by RT-PCR using nine sequential primer pairs covering 3'UTR of *WEE1* and encompassing TCS. RNA gained from CLIP was reversely transcribed and triplicate RT-PCR is exemplarily shown for primers 8 and 9 in gel electrophoresis together with positive control.

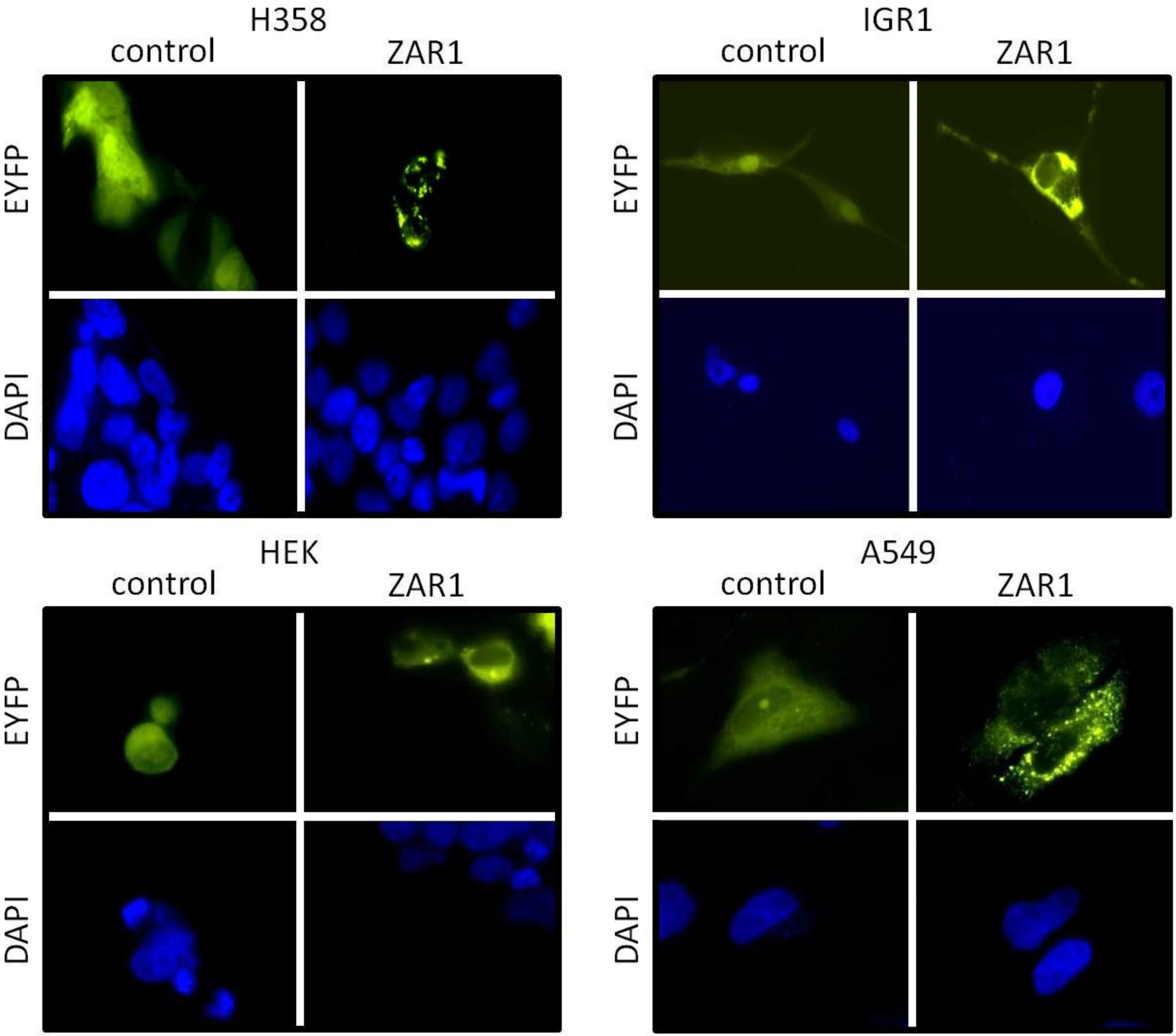

Suppl. Fig. 7: ZAR1 is cytosolic. ZAR1-EYFP is found exclusively within the cytosol, which is cell line independent. H358, IGR1, HEK and A549 cells were grown on glass slides, transfected with empty-EYFP and ZAR1-EYFP and formaldehyde fixed after 24h. DNA was stained with DAPI and slides were embedded in Mowiol before microscopy (63x).

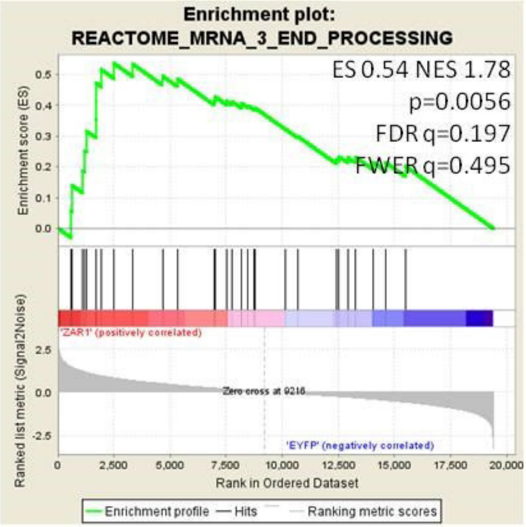

Suppl. Fig. 8: Reexpression of ZAR1 alters the transcriptome and reveals association with mRNA 3'end processing. Enrichment plot from RNA micro array of ZAR1 overexpression reveals association with 'mRNA 3'end processing' by GSEA analysis and according heatmap with annotated genes is shown for ZAR1 and EYFP overexpression after 30h in HCT116 cells.

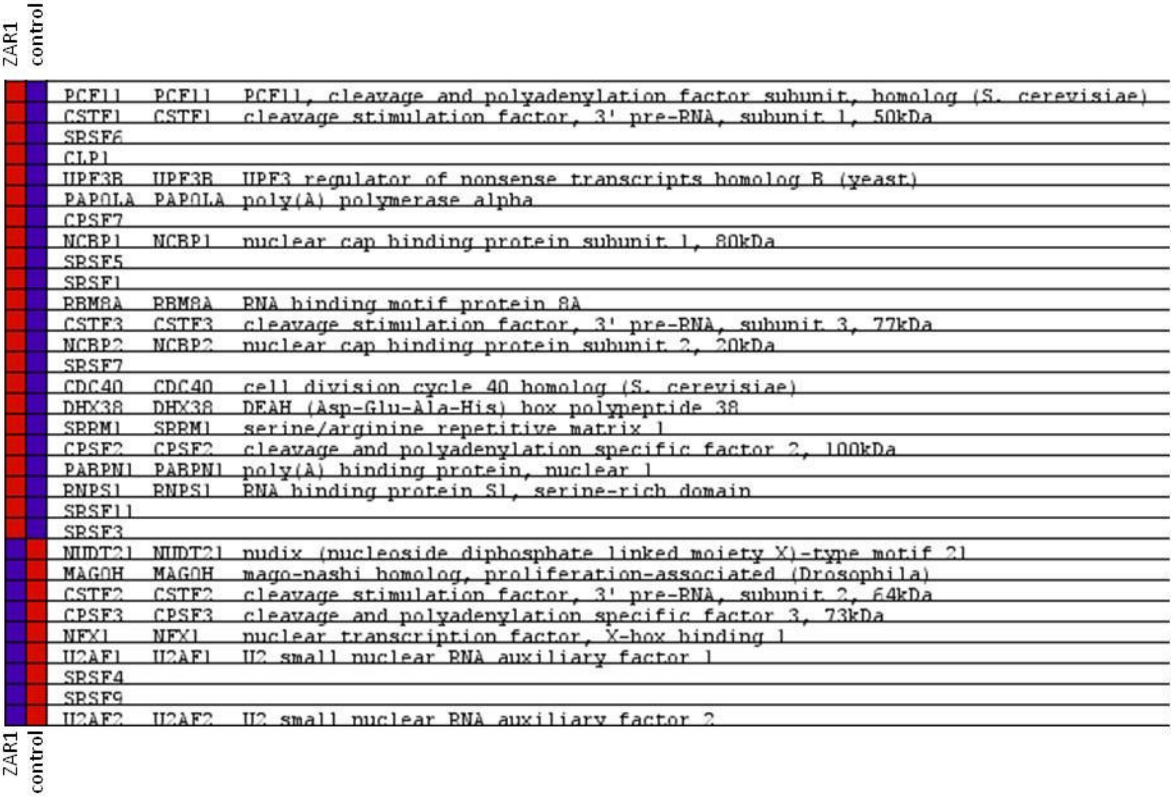

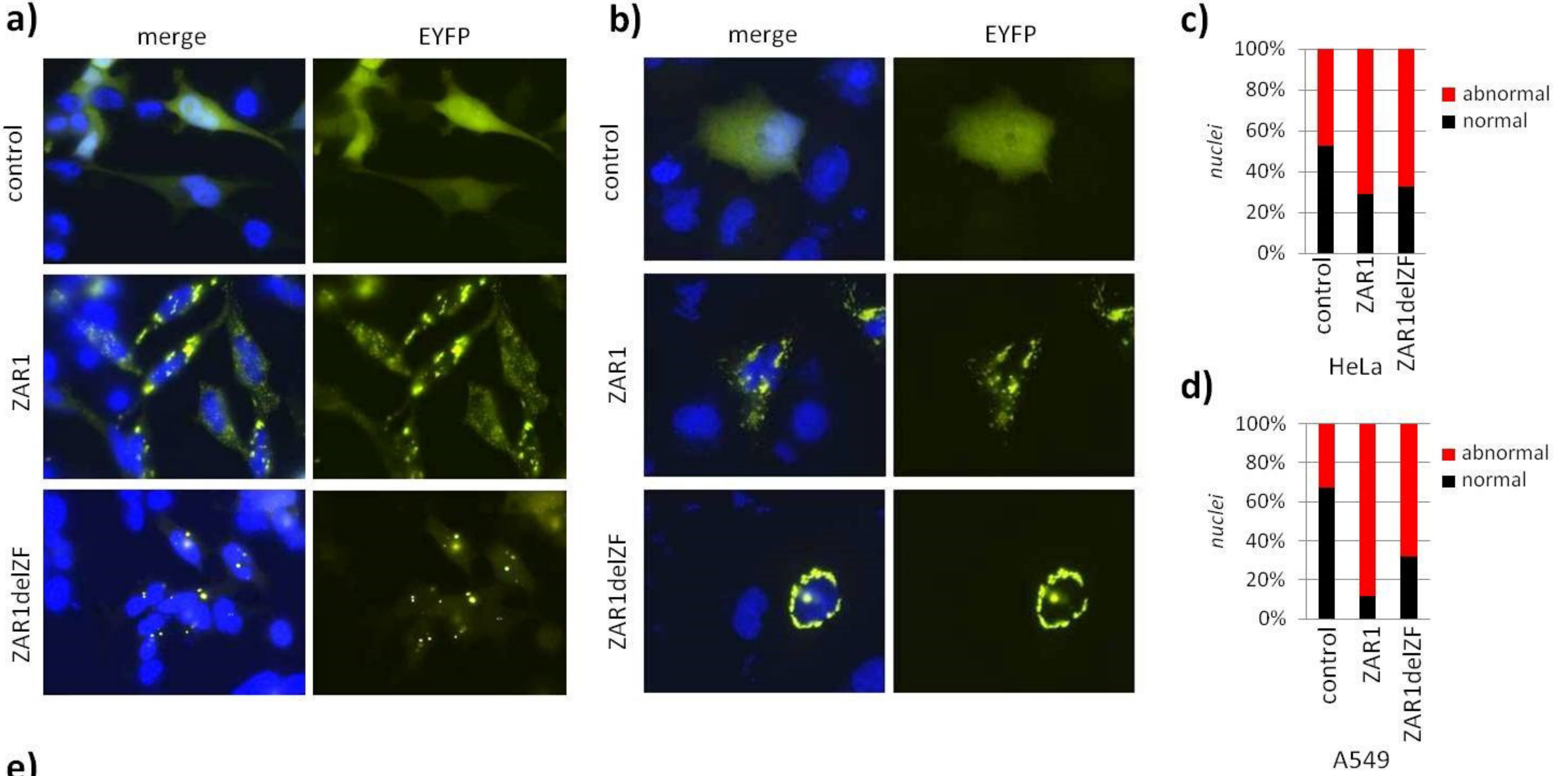

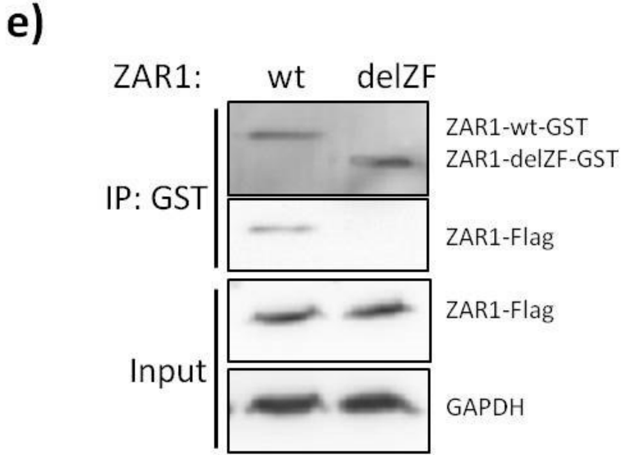

Suppl. Fig. 9: ZAR1 function depends on its zinc finger. a) ZAR1-EYFP, ZAR1delZF-EYFP (deleted zinc finger) and EYFP-empty were overexpressed for 24h in **a)** HeLa and **b)** A549. Cells were formaldehyde fixed, DNA was DAPI stained (blue) and embedded in Mowiol before microscopy (63x). Malformation of nuclei morphology upon overexpression of ZAR1 was counted (>100 nuclei) and summarised for **c)** HeLa and **d)** A549. **e)** ZAR1 zink finger dependent dimer formation of ZAR1 after overexpression of ZAR1-GST (wildtype wt or Zink finger deletion mutant delZF) together with ZAR1-Flag followed by GST pulldown, SDS-Page separation and western blotting.

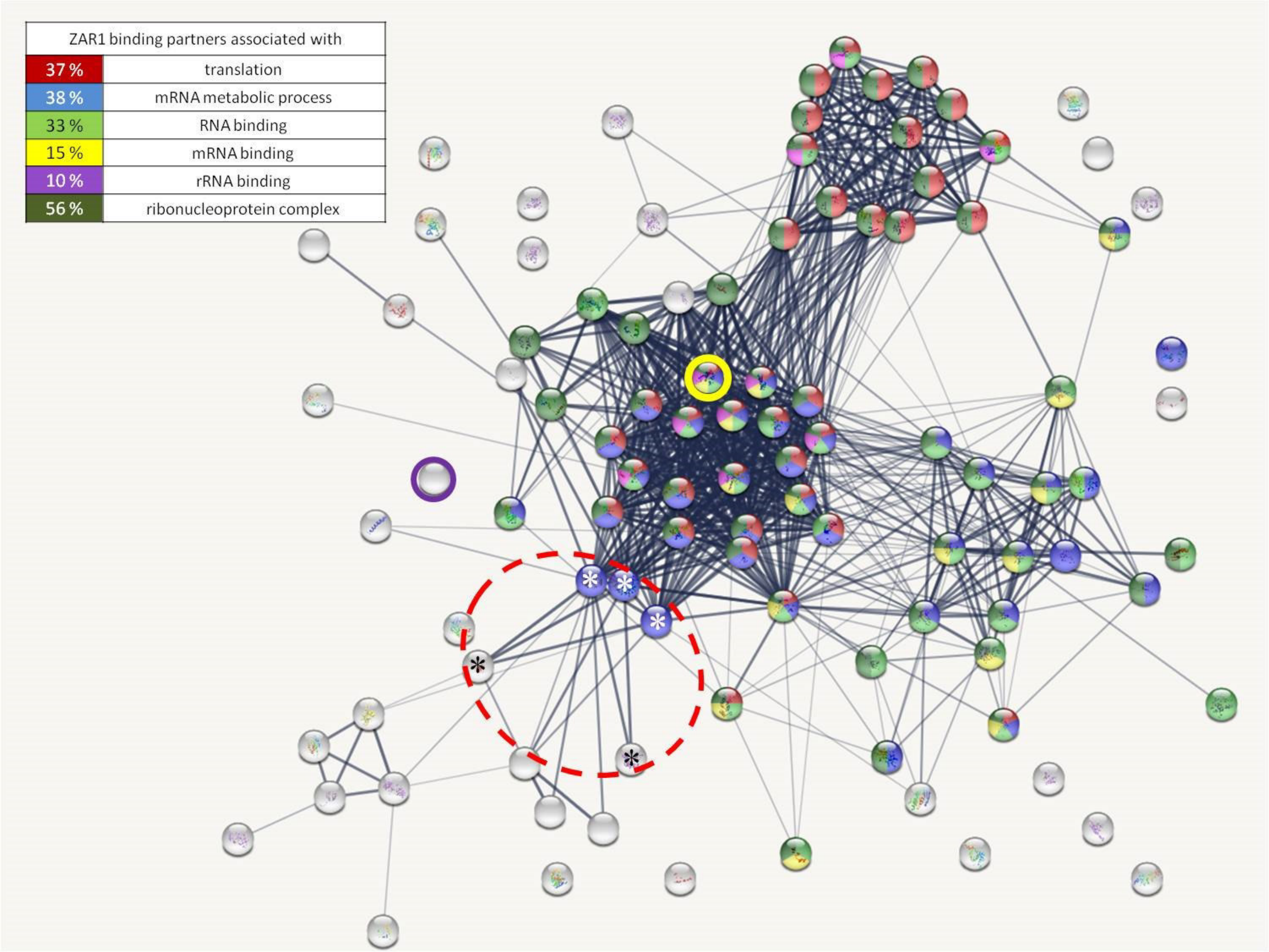

Suppl. Fig. 10: ZAR1 binding partner GO-term association overview. ZAR1 binding partner association with GO terms reveals significant cluster formation and is shown colour coded (red: translation 1.3E-33, blue: mRNA metabolic process 1.7E-26, light green: RNA binding 7.3E-18, yellow: mRNA binding 2.2E-11, purple: rRNA binding 1.7E-10 , dark green: ribonucleoprotein complex 2.2E-49). Total number of nodes is 101 with a PPI enrichment p-value <1e-16. Line thickness (grey) indicates strength of data support. ZAR1 is circled in purple.Asterisks indicate network partners that are unaffected by deletion of the ZAR1 zinc finger and circled in red. Yellow circle marks strongest binding partner of ZAR1, that is lost upon zinc finger deletion.

ZAR1delZF binding partners associated with		GO-term	GO-term	Count in gene set/total MS count	Total GO gene set	FDR
29 %	mitotic cell cycle phase transition	Biological Process	GO:0044772	5/17	254	7.0E-04
24 %	peptidyl-serine dephosphorylation		GO:0070262	4/17	12	4.7E-07
-	-	Molecular Function	-	-	-	-
-	-		-	-	-	-
-	-		-	-	-	-
24 %	protein phosphatase type 2A complex	Cellular Component	GO:0000159	4/17	17	3.2E-07

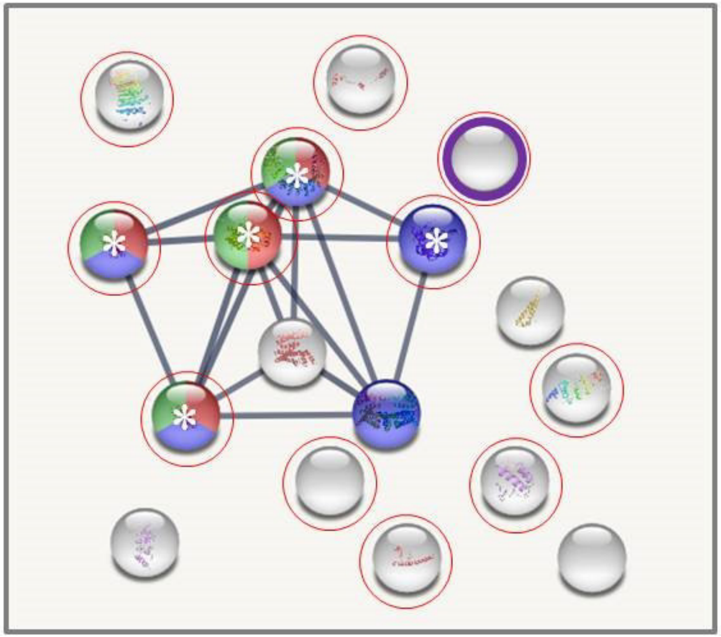

Suppl. Fig. 11: ZAR1 binding partner GO-term association overview. ZAR1delZF binding partner association with GO terms reveals minimal cluster formation and is shown colour coded (blue: mitotic cell cycle phase transition 7.0E-04, red: peptidyl-serine dephosphorylation 4.7E-07 and green: protein phosphatase type 2A complex 3.2E-07). Total number of nodes is n=17 incl. ZAR1. Line thickness (grey) indicates strength of data support. ZAR1 is circled in purple. Red circles indicate presence also in ZAR1wt associated partners. Asterisks indicate network partners that are also found in ZAR1wt network and are zinc finger independent.

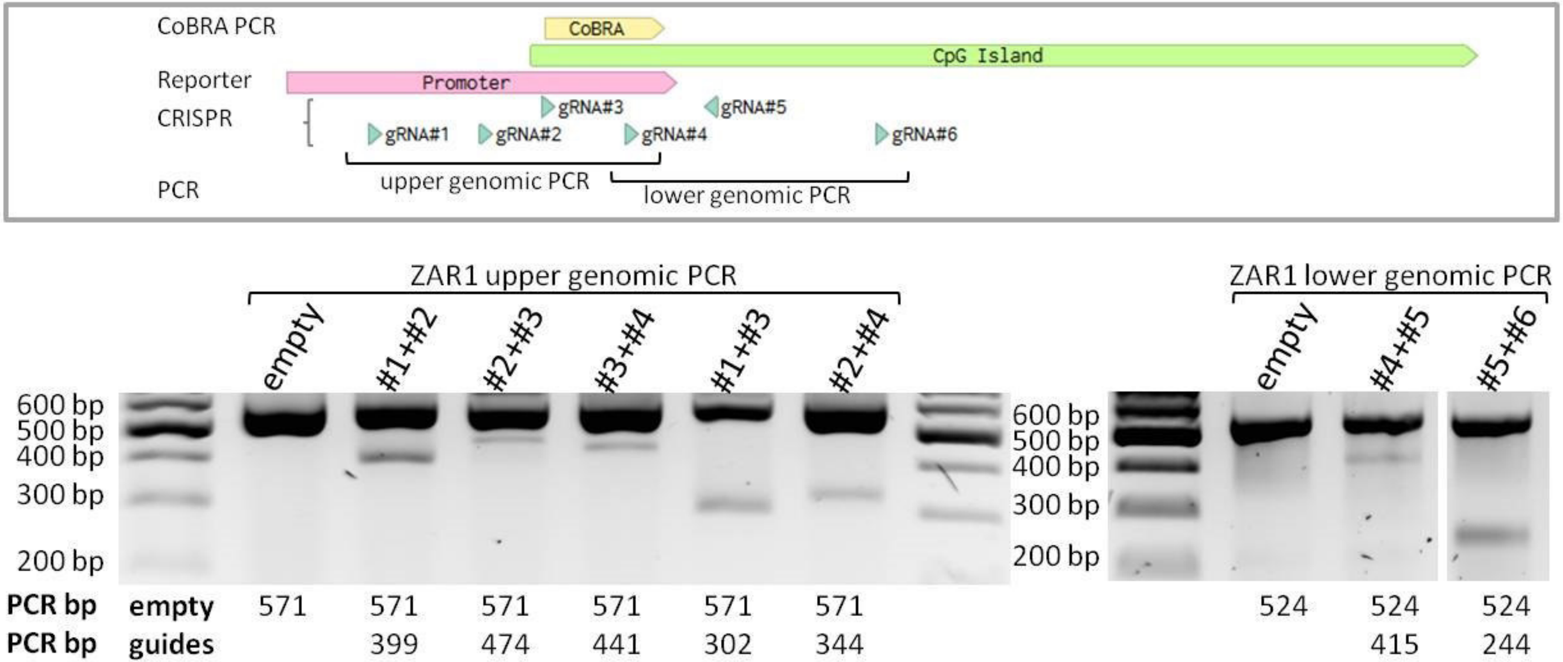

Suppl. Fig. 12: Targeting the ZAR1 genomic region with CRISPR ZAR1 guide RNA oligos. Targeting of the *ZAR1* genomic region with combined ZAR1 RNA guides is resulting in expected genomic deletion by genomic PCR for ZAR1 (shown for various guides combination and PCR primers were designed to flank guides). HEK cells were transfected with indicated guide combination and DNA was isolated after 24h. Confirming the ZAR1 genomic binding ability of the designed CRISPR ZAR1 guide RNAs.

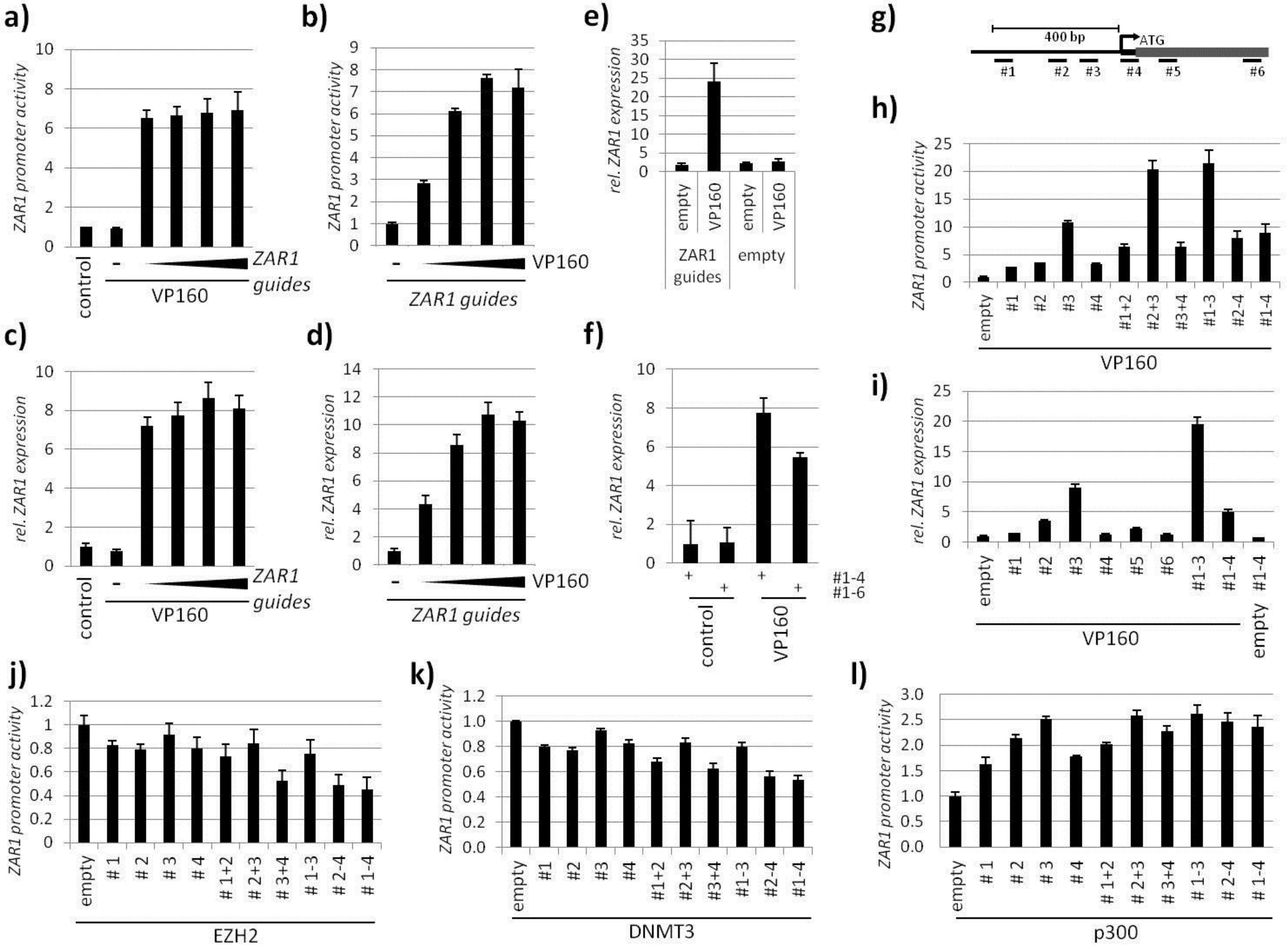

Suppl. Fig. 13: Effective epigenetic editing of ZAR1 with distinct RNA guide combinations upstream the TSS. a+c) Optimal dose of ZAR1 guides and **b+d)** epigenetic modulator VP160 were determined by ZAR1 promoter driven luciferase expression as well as ZAR1 endogenous expression. **a)** ZAR1 promoter assay upon overexpression of ZAR1 guide amounts (0, 0.35, 0.7, 1 or 1.4µg) together with constant VP160 activator (or empty control) and **b)** upon overexpression of VP160 amounts (0, 5, 50, 500ng, 1µg) together with constant ZAR1 guides. **c)** Endogenous *ZAR1* activation upon overexpression of ZAR1 guide amounts (0.35, 0.7, 1 or 1.4µg) together with constant VP160 activator (or emtpy control) and **d)** upon overexpression of VP160 amounts (0, 50ng,0.5µg, 1µg, 3µg) together with constant ZAR1 guides. **e)** Endogenous *ZAR1* activation upon overexpression of VP160 and ZAR1 guides, but not unguided or by VP160 alone. **f)** Endogenous *ZAR1* expression is most effective by ZAR1 guides upstream of TSS (#1-4). **g)** Depiction of ZAR1 guides relative to TSS and ATG with 400bp scale. **h)** Determination of optimal ZAR1 guides by ZAR1 promoter assay upon overexpression of indicated guide combinations together with VP160 and **i)** according results for endogenous *ZAR1* activation by combinations of ZAR1 guides. **j-l)** Determination of optimal ZAR1 guides by ZAR1 promoter assay upon overexpression of indicated guide combinations together with either EZH2, DNMT3 or p300.

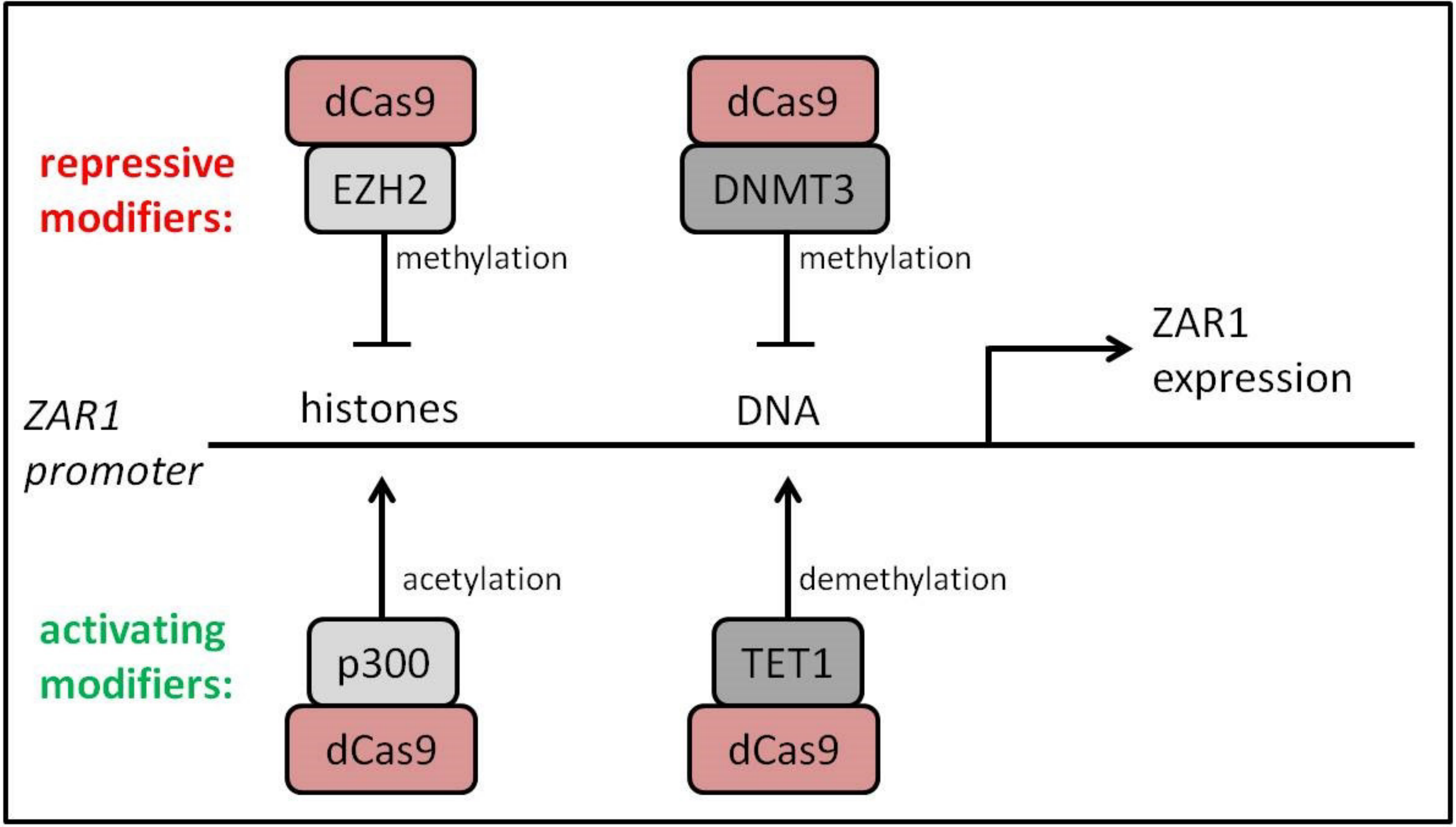

Supplementary Fig. 14: Schematic representation of epigenetic modifiers by CRISPR dCas therapy on ZAR1 The ZAR1 promoter is targeted by RNA guides specific for the intended region of the ZAR1 promoter. The CRISPR RNA guides are incorporated in dCas9 (red), which is nuclease deficient, and recruits the epigenetic modifiers (grey, EZH2, DNMT3, p300 or TET1) to ZAR1. The epigenetic modifiers act on ZAR1 by methylation/acetylation of histones or methylation/demethylation of DNA. This modification then modulates ZAR1 expression.
